# The genome-scale interplay amongst xenogene silencing, stress response and chromosome architecture in *Escherichia coli*

**DOI:** 10.1093/nar/gku1229

**Published:** 2014-11-27

**Authors:** Rajalakshmi Srinivasan, Vittore Ferdinando Scolari, Marco Cosentino Lagomarsino, Aswin Sai Narain Seshasayee

**Affiliations:** 1 National Centre for Biological Sciences, Tata Institute of Fundamental Research, GKVK, Bellary Road, Bangalore 560065, India; 2 Manipal University, Manipal 576104, India; 3 Genomic Physics Group, UMR 7238 CNRS Microorganism Genomics, UPMC, Paris, France; 4 Sorbonne Universités, UPMC Univ Paris 06, UMR 7238, Computational and Quantitative Biology, 15 Rue de l’École de Médecine Paris, France; 5 CNRS, UMR 7238, Paris, France

## Abstract

The gene expression state of exponentially growing *Escherichia coli* cells is manifested by high expression of essential and growth-associated genes and low levels of stress-related and horizontally acquired genes. An important player in maintaining this homeostasis is the H-NS-StpA gene silencing system. A *Δhns-stpA* deletion mutant results in high expression of otherwise-silent horizontally acquired genes, many located in the terminus-half of the chromosome, and an indirect downregulation of many highly expressed genes. The *Δhns-stpA* double mutant displays slow growth. Using laboratory evolution we address the evolutionary strategies that *E. coli* would adopt to redress this gene expression imbalance. We show that two global gene regulatory mutations—(i) point mutations inactivating the stress-responsive sigma factor RpoS or σ38 and (ii) an amplification of ∼40% of the chromosome centred around the origin of replication—converge in partially reversing the global gene expression imbalance caused by *Δhns-stpA*. Transcriptome data of these mutants further show a three-way link amongst the global gene regulatory networks of H-NS and σ38, as well as chromosome architecture. Increasing gene expression around the terminus of replication results in a decrease in the expression of genes around the origin and vice versa; this appears to be a persistent phenomenon observed as an association across ∼300 publicly-available gene expression data sets for *E. coli*. These global suppressor effects are transient and rapidly give way to more specific mutations, whose roles in reversing the growth defect of H-NS mutations remain to be understood.

## INTRODUCTION

A variety of parameters orchestrate the gene expression state of a cell. For example, several global transcription factors and chromatin structuring proteins—together with a constellation of local transcription regulators—ensure maintenance of gene expression homeostasis. In exponentially growing *Escherichia coli* cells, many essential and growth-associated genes are expressed at high levels, whereas stress-related and horizontally acquired genes are maintained in low or silent expression states. Global disruption of this balance between the expression levels of growth-associated and stress-related genes, by mutations in regulatory proteins, can be expected to adversely affect fitness. Whether and how a bacterial cell adapts to such a loss of fitness is a question that is of considerable interest, more so in the light of evidence that gain and loss of transcriptional regulatory functions is not uncommon (1).

A single chromatin-structuring gene-silencing system (2–4), whose central players are the protein H-NS and its homologue StpA (5), is responsible for a global shutdown of A+T-rich horizontally acquired genes in *E. coli*. Loss of this gene silencing system dramatically upsets the gene expression balance of the cell, by causing otherwise silent loci to be expressed at exceptionally high levels (6). This might—at least in part—be due to pervasive transcription (7) from A+T-rich sequence stretches to which H-NS and StpA bind. A *Δhns-stpA* double mutant of *E. coli* K12 also suffers from a severe reduction in growth rate (8), which might emerge from the ‘cost’ associated with uncontrolled expression of transcriptionally silent genes (6). Being a regulator of horizontally acquired genes, H-NS targets different gene functions even across closely related bacteria (e.g. a large proportion of H-NS targets in *E. coli* are not conserved in *Salmonella* and vice versa (9)), making its regulatory network dynamic, and subject to disruption (10) by rampant horizontal gene acquisition.

We exploited these characteristics of the *Δhns-stpA* double mutant to investigate the evolutionary trajectories that help *E. coli* redress the global disruption of its gene expression state, using *in vitro* laboratory evolution experiments, analysed using deep sequencing of genomes and transcriptomes.

## MATERIALS AND METHODS

### Strains and general growth conditions

The *E. coli* variants used in the work are following: *E. coli K-12 MG1655* wild type (CGSC #6300); MG1655 *Δhns-stpA (Δhns-stpA::kan^r^*); *Δhns-stpA-rpoS (ΔstpA-hns-rpoS::kan^r^*). HS100 refers to the population of *Δhns-stpA* mutant evolved for ∼100 h under laboratory conditions and HS250 to those evolved for ∼250 h. *ori^2^* refers to a single colony isolated from HS100, showing a duplication of ∼2-Mb region around the origin, and *rpoS^mut^* to a single colony from HS100, with an inactivating point mutation in *rpoS*. Luria broth was used for normal growth, 50 μg/ml of ampicillin or kanamycin was used as per requirement

### Construction of *E. coli* K12 MG1655 knockouts

All gene deletions were achieved by the Lambda Red recombination system, described by Datsenko and Wanner using primers pKD46 and pKD4 or pKD13 (11). Knockout strains generated by this method were selected on LB Kanamycin (50 mg/ml) plates and deletion was confirmed by polymerase chain reaction (PCR) using specific primers. Double knockouts were also generated by the same method after removing the kanamycin cassette using pCP20.

### Laboratory evolution experiments

The laboratory evolution experiment was carried out for two parallel populations of an overnight grown culture, revived from the frozen stock made from a single colony of *Δhns-stpA*. Cells were grown in 24-well plates, shaking at 200 rpm, until late exponential phase and diluted by a factor of 1:50 into fresh Luria-Bertani (LB) broth. Glycerol stocks were made at the end of every passage. HS100 and HS250—populations evolved for ∼100 and 250 h, respectively—were selected for genome sequencing. For single colony genome sequencing, a sample of HS100 or HS250 cells was plated on LB agar, and single colonies picked; glycerol stocks were made for all the single colonies isolated. This procedure is represented by a schematic figure in Supplementary Figure S1.

### Growth curves

Overnight grown culture was inoculated in fresh LB to a 1:100 ratio and the growth of cells monitored by measuring the optical density at 600 nm. All these growth experiments were performed in 96-well plates, incubated at 37°C in a plate reader (Tecan, infinite® F200 PRO) with constant shaking. OD600 was measured every ∼16 min. Each growth curve trial comprised two biological and six technical replicates.

### Whole genome sequencing

Genomic DNA was isolated from *Δhns-stpA* and evolved strains using SIGMA GenElute™ Bacterial Genomic DNA Kit (Cat. No. NA2120) using the manufacturer's protocol. Sequencing library was prepared for Illumina sequencing and the quality of the libraries checked using Agilent Bioanalyzer at the genomics facility, Centre for Cellular and Molecular Platforms (C-CAMP), Bangalore. DNA isolated from HS100 and HS250 populations was sequenced for 50 cycles from one end, and that from the single colonies sequenced for 100 cycles from both ends. The sequencing was performed on the Illumina HiSeq1000 platform at C-CAMP. Raw data have been deposited with NCBI-SRA under the accession number SRP043310.

### Mutation calling

50-mer single-end reads obtained from HS100 and HS250 populations were mapped to *E. coli* K12 MG1655 genome (NC_000913, v2) using Burrows Wheeler Aligner (BWA) (12) using the -q 20 parameter, sorted and indexed with SAMtools (13). Single Nucleotide Polymorphisms (SNPs) and indels were detected using VarScan (14). SNPs supported by more than 20% of reads from the population genome sequencing experiments are listed in Supplementary Figure S2 and in Supplementary Table S1; these lists include only those mutations which were not detected in the parental genome of *Δhns-stpA*. SNPs and indels were identified from the single colony genome sequencing data using the BRESEQ pipeline (15), which uses Bowtie (16) for sequence alignment; these results can be browsed at http://bugbears.ncbs.res.in/hns_evol. SNPs identified using this analysis were validated by Sanger sequencing using specific primers.

Read coverage across the genome was calculated for non-overlapping windows of 200 nt each using custom scripts and normalized by the mode of the distribution across these bins as described earlier (6). Coverage is defined by the number of sequencing reads that map to a region on the chromosome, and is represented on the log, base 2 scale.

### RNA extraction and mRNA enrichment

For RNA extraction, the overnight cultures were inoculated in 100 ml of fresh LB to bring the initial Optical Density (OD) of the fresh culture to 0.03 and the flasks were incubated at 37°C with shaking at 200 rpm. Two biological replicates were performed for each sample. Samples were collected at the mid-exponential phase (OD600 ∼ 0.5 for *Δhns-stpA*; 0.7 ∼ 0.8 for *rpoS^mut^, Δhns-stpA-rpoS* and *ori^2^*; 0.9 for the wild type). Protocols, based on those recommended by the manufacturer for the TRIzol (Invitrogen) bacterial RNA isolation kit, were used for RNA isolation as described previously (6). Libraries were prepared for RNA-seq and sequenced for 50 cycles from one end on the Illumina HiSeq 1000 platform, following manufacturer's recommendations, at C-CAMP. Raw data have been deposited with NCBI-SRA under the accession number SRP043518.

### Transcriptome data analysis

50-mer single-end sequence reads were mapped to the *E. coli K12 MG1655* genome using BWA (12). Gene annotations were obtained from the Ecocyc database (17). The number of reads falling within each gene was calculated based on the base position to which first nucleotide of the read was mapped. A matrix containing the read count for each gene across the sequenced samples was fed into the Bioconductor (http://www.bioconductor.org) package EdgeR (18) for analysis of differential expression. The genes that are differentially expressed by at least 2-fold with a *P*-value of 0.00001 were considered for further analysis.

Differential expression in the *ori^2^* strain was calculated differently. Here, the mode of the distribution of per-gene read counts (6) across only the non-amplified portion of the genome was used as a normalization factor. This was performed for *ori^2^* as well as the strains against which *ori^2^* was compared: the parental *Δhns-stpA* and the wild type. Read coverage distributions shown in Supplementary Figure S3 indicate that this normalization factor does not affect the gene expression measures for strains not carrying the amplification. The normalized read counts were processed for differential expression using the LIMMA pipeline (19). Supplementary Figure S4 shows a comparison of fold changes obtained using the above pipeline as well as the standard EdgeR procedure; this shows that there is a tight correlation between the two, except that the fold change estimates are consistently off by a factor of 2^∼0.3^ across most genes.

### Analysis of publicly-available microarray data

Publicly-available gene expression (transcriptome) data for *E. coli* were downloaded from the M3D database (http://m3d.mssm.edu; (20)). This comprises Robust Multi-array Average (RMA)-normalized gene expression measurements from Affymetrix microarrays across ∼300 conditions. Pearson correlation coefficients (PCC) between pairs of gene expression vectors were calculated using a combination of PERL and R scripts. High positive PCC between a pair of genes indicates that their expression levels increase or decrease similarly across the conditions included in the data set. A high negative PCC, on the other hand, indicates opposing gene expression patterns for the two genes (referred to as anti-correlation): an increase in the expression level of one gene is met with a corresponding decrease in that of the other. The chromosome was binned into segments of 100 kb each, and each gene assigned to a segment based on its position on the genome. For each pair of segments, the number of genes with correlated (PCC > = 0.5) or anti-correlated (PCC < = -0.5) gene expression vectors was computed and plotted as a matrix using the ‘matrix2png’ web server (www.chibi.ubc.ca/matrix2png).

### Validation of RNA sequencing data using reverse transcriptase-PCR

Selected RNA sequencing results—from comparisons involving *rpoS* mutants—were validated using quantitative reverse transcriptase-PCR (RT-PCR). mRNA was isolated from the cells collected at the same time points as for RNA sequencing using TRIZOL, followed by DNase treatment. mRNA after DNase treatment was precipitated using ammonium chloride as described in the MICROBExpress RNA purification kit and used for the RT-PCR. RT-PCR reactions were carried out using the reagents provided with the Takara one step RT-PCR kit using manufacturer's instructions. 16S RNA gene, *rrsA*, was used as an internal control. The outcome of these experiments is presented in Supplementary Table S2 (resulting in a Spearman correlation coefficient > 0.9 between fold-changes measured by RT-PCR and that by RNA-seq), and the primers used in Supplementary Table S3.

### Catalase assay

10 μl of overnight grown culture was spotted on an LB agar plate and incubated at 37°C for 24 h, after which 10 μl of H_2_O_2_ was added. The cells with active σ38 effervesce almost immediately, whereas those with inactive σ38 show delayed effervescence. Approximate time taken for the effervescence to start was measured and used as a proxy for σ38 activity.

## RESULTS AND DISCUSSION

### Disruption of gene expression homeostasis in *Δhns-stpA*

**Figure 1.**
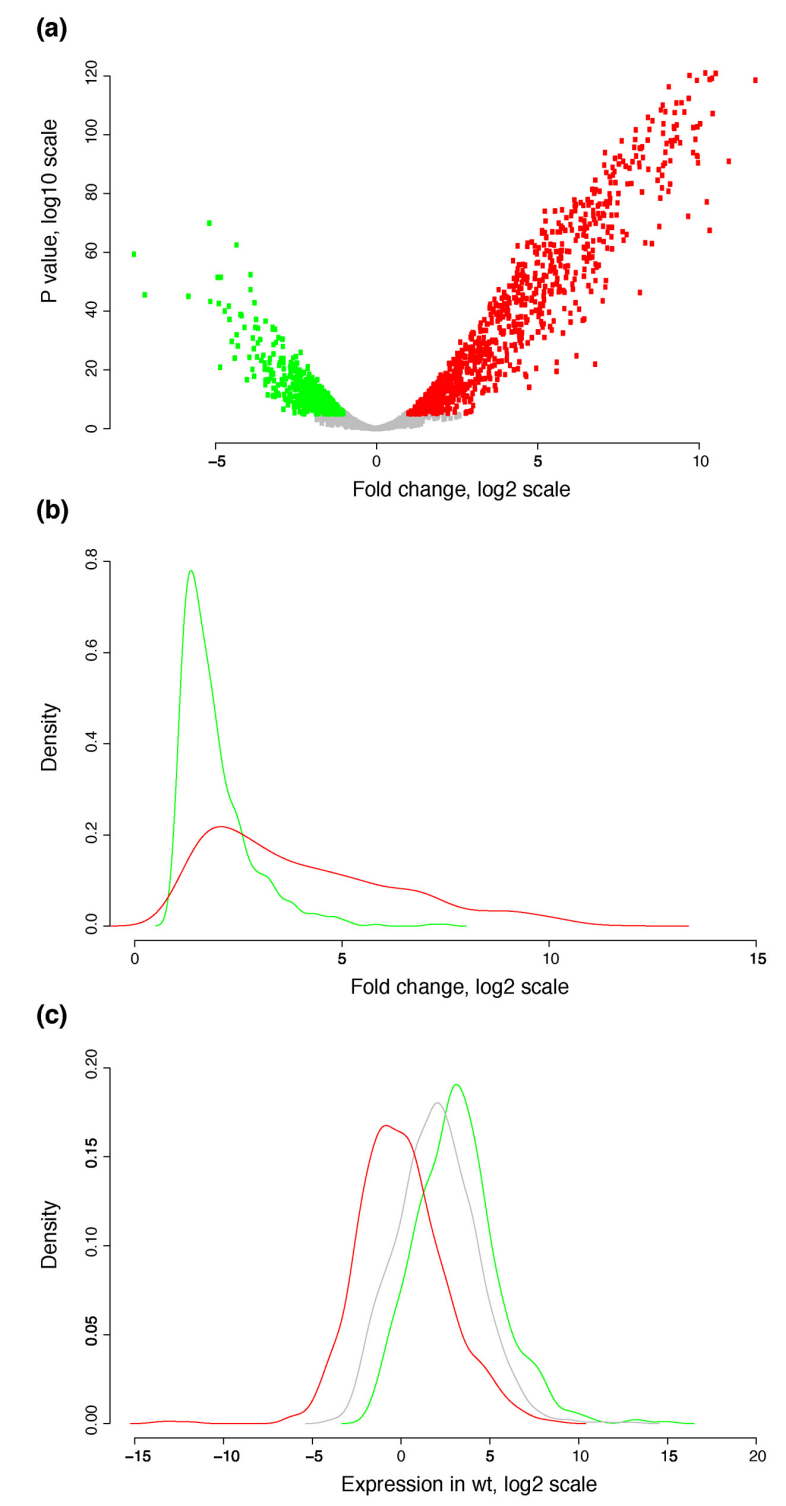
Large-scale disruption of gene expression states in a *Δhns-stpA* mutant. (**a**) A volcano plot of fold change (log_2_ scale) in expression levels between the *Δhns-stpA* double mutant and the wild type on the x-axis, and the *P*-value (log_10_ scale) for the measured fold change on the y-axis. Red dots show genes that are upregulated in the mutant relative to the wild type and green dots represent genes that are downregulated in the mutant. (**b**) Distributions of absolute fold changes (log_2_ scale) for genes that are up (red) or downregulated (green) in *Δhns-stpA* relative to the wild type. (**c**) Wild-type expression levels of genes that are up (red) or downregulated (green) in *Δhns-stpA* relative to the wild type; control genes, which are not significantly differentially expressed are represented by the grey coloured distribution.

We had previously constructed a double *Δhns-stpA* deletion mutant in *E. coli* K12 MG1655 using homologous recombination (6). The transcriptome of this strain—relative to the wild type—during mid-exponential phase in LB medium exhibits an increase in the expression of many A+T-rich genes, consistent with the known role of H-NS as a global gene silencer. This strain also displays a considerably smaller growth rate than the parental wild-type strain, and those of the two single mutants *Δhns* and *ΔstpA* (6,8). Here we generated new, higher-coverage RNA-seq data for *Δhns-stpA*, which are consistent with the above-described work. Here, we additionally report that a large number of genes are also downregulated. In these data, ∼920 genes were upregulated in *Δhns-stpA* when compared to the wild type, whereas ∼650 were downregulated in the mutant (Figure 1a). A significant proportion of upregulated genes are also known to be bound by H-NS (∼67%; *P* < 10^−10^; Fisher's exact test), as deduced from ChIP-seq data identifying the *in vivo* binding sites of H-NS on the *E. coli* chromosome during the mid-exponential phase of growth (9,21). In contrast, a much smaller percentage of the downregulated genes is bound by H-NS. In fact, the chance that an H-NS-bound gene is downregulated in *Δhns-stpA* is less than expected by random chance (∼17%; *P* ∼ 10^−6^; Fisher's exact test). The fold change of differential expression per downregulated gene is significantly less than that for upregulated genes (*P* < 10^−10^; Wilcoxon test; Figure 1b). Genes that are downregulated in the *Δhns-stpA* mutant showed a higher median wild-type expression level than those that are not (*P* < 10^−10^; Wilcoxon test; Figure 1c). The downregulation of many genes with high expression levels may be an indirect consequence of the redirection of the limited pool of RNA polymerase molecules (22) to high level transcription of otherwise silent genes. This compensation might be consistent with the observation that the distribution of gene expression from ∼2000 *E. coli* promoters is invariant across conditions (23), even though the location of a given gene within the distribution may differ. Thus, loss of a global gene silencing system in *E. coli* not only results in the upregulation of its direct targets but also indirect downregulation of a large number of otherwise highly expressed genes, indicating a global disruption of gene expression homeostasis.

### *In vitro* laboratory evolution of *Δhns-stpA*

We asked whether and how the bacterium would adapt to such a global upset of its gene expression state. Towards this, we performed *in vitro* laboratory evolution experiments (Supplementary Figure S1). In these experiments, two parallel populations of *Δhns-stpA*—derived from the same colony, isolated and frozen soon after the experimental deletion of *hns* and *stpA*—were grown in LB medium in 24-well plates under batch conditions. These experiments set up a dynamic ‘survival of the fittest’ competition in liquid cultures, selecting for bacterial variants that are fitter than the starting parent bacterium, leading to the discovery of suppressors of the growth defect of *Δhns-stpA*. Subculturing into fresh medium was performed towards the end of exponential growth or early in stationary phase. This ensures that selection will be for increasing growth rate, and not be influenced by stationary phase survival.

**Figure 2.**
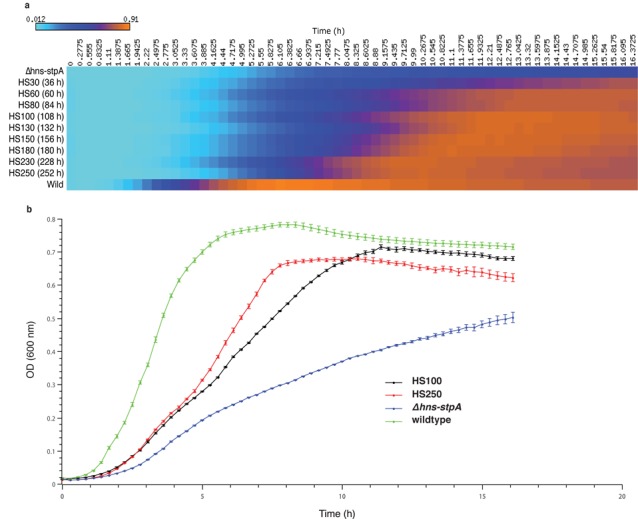
Suppression of the growth defect of *Δhns-stpA* during the course of a laboratory evolution experiment. (**a**) A heatmap representation of growth curves of bacterial populations obtained after the specified hours of evolution. Each row represents a snapshot of the evolution experiment, and each column a time point during a single batch culture. The snapshots labelled HS100 (∼108 h, spread across nine serial batch cultures) and HS250 (252 h, across 21 serial batch cultures) were selected for population genome sequencing. The wild-type (bottom row) and the *Δhns-stpA* parent (top row) are also indicated. The colour in each cell is indicative of OD_60_, with cyan representing the lowest values and orange the highest ones. (**b**) Growth curves of HS100 (black), HS250 (red), the wild-type (green) and the *Δhns-stpA* parent (blue). This is a zoom-in—in standard representation—of the two frames from (a), which have been subjected to sequencing analysis in this study. The error bars show the standard error across two biological and six technical replicates.

We observed a rapid, but steady increase in growth rate over the evolution experiment which together covered only a period of ∼250 h, spread across ∼20 serial batch cultures (Figure 2a). For further analysis we studied the characteristics of the genomes of two frames, one from the 100-h population and the other from the 250-h population (HS100 for the ∼100 growth-hour frame; HS250 for the ∼250 growth-hour frame; Figure 2b), and compared them with those of the parent (*Δhns-stpA*). The discussion in this paper will be largely restricted to our analysis of HS100, an early time point in the evolution of *Δhns-stpA* towards higher fitness.

### Growth recovery by σ38 inactivation

We performed deep sequencing (50-mer from a single end, Illumina HiSeq-1000) of populations—one for the parental population *Δhns-stpA*, and one for each of the two evolved lines for HS100 and HS250, giving ∼250–380-fold coverage of the haploid genome per sample. Note that these sequences are not derived from single clones, but from populations. Therefore, subject to sampling errors (24), these data help identify most variants that are present above a certain level in the population. The proportion of reads supporting a variant could be expected to be roughly indicative of its prevalence in the population.

We first performed an analysis of point mutations and short indels in HS100 in comparison to *Δhns-stpA* (Supplementary Figure S2; Supplementary website: http://bugbears.ncbs.res.in/hns_evol). This showed that the population is genetically heterogeneous, with differences between the two populations in the mutations accrued. All mutations detected were present only in a sub-population, indicating the presence of multiple trajectories. We do not discuss each of these mutations here, but present selected observations.

About 40% of the sequence reads from one of the two populations displayed a polymorphism in the *rpoS* gene, encoding the σ38 factor (RpoS) responsible for the general stress response of *E. coli*. We performed paired-end sequencing (100-mer from each end) of the genomes of 16 colonies, all derived from the HS100 population carrying the mutation in *rpoS*. Bacteria from each of the 16 colonies showed higher growth in liquid LB medium when compared with *Δhns-stpA* (Supplementary Figure S5). Analysis of single-nucleotide variations in these data, using the BRESEQ pipeline or otherwise from first principles, revealed the presence of different polymorphisms in *rpoS* (Supplementary Figure S2; Supplementary website: http://bugbears.ncbs.res.in/hns_evol). For example, several mutants carried a STOP codon at position E43 or Y61 of the σ38 amino acid sequence, indicating an inactivating mutation. Together, eight colonies (nine colonies, including one with a mutation supported by <85% of reads), carried at least one mutation in RpoS. That these mutations lead to inactivation of the σ38 regulon was verified by a simple test for catalase activity, in which a patch of cells with active σ38 effervesce rapidly on addition of H_2_O_2_, whereas those lacking functional σ38 do not (data not shown); this emerges from the dependence of the expression of catalase gene expression on σ38. To further validate the role of σ38 inactivation in increasing the growth rate of *Δhns-stpA*, we constructed a *ΔrpoS-hns-stpA* triple deletion mutant. This mutant shows a higher growth rate than *Δhns-stpA*, to a level comparable to that of the HS100 clones (Supplementary Figure S6).

### A large amplification and growth recovery

**Figure 3.**
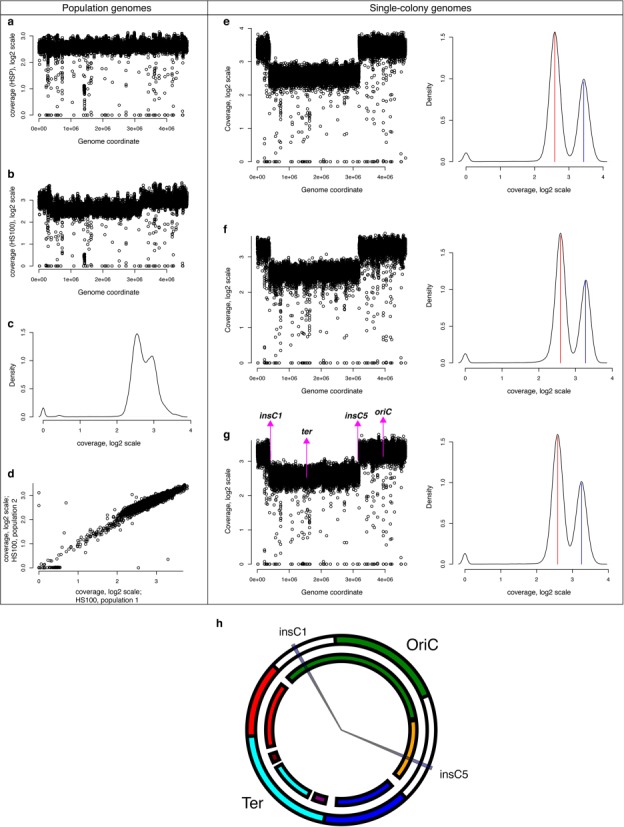
Amplification of ∼40% of the *E. coli* chromosome around the origin of replication. The read coverage, on log_2_ scale, as a function of genome coordinate is plotted for (**a**) *Δhns-stpA* and for (**b**) the evolved population HS100. (**c**) Distribution of read coverage across the chromosome for HS100, directing the reader to its bimodal shape. (**d**) Correlation in coverage between the two HS100 populations sequenced. (**e, f**) Read coverage—as a function of genome coordinate (left) and as a distribution (right)—for the three HS100 clones carrying the duplication. Note the strongly bi-modal nature of the read coverage distributions, where the two modes are marked by vertical lines (low coverage in red and high coverage in blue); these approximate the average coverage of the duplicated and the non-duplicated segments of the chromosome on a log_2_ scale. (**h**) Circular plot of the chromosome, indicating the following key elements: insC1 and insC5, which are the end points of the duplication; the origin (OriC) and the terminus (Ter) of replication. The outer circle marks the major structural domains of the chromosome as defined by the elegant definition of macrodomains (outer circle) by Valens *et al.* (39), where the unstructured elements are in white. The plot was drawn using the NuST web server (40). The inner circle defines ‘chromosomal sectors’, which represent domains showing correlations between gene expression and codon usage (41).

We performed a detailed analysis of the read coverage distribution of the HS100 population and compared it with that of *Δhns-stpA*. The read coverage is a measure of the relative abundance or copy number of a particular locus in a genome. In the *Δhns-stpA* population, we see a nearly uniform read coverage across the genome (Figure 3a), with exceptions including the *rac* prophage region (Supplementary Figure S7), which appears to have been deleted at least in a subset of the population (25), and various other repetitive elements where unique read mapping is not possible.

The two HS100 populations however showed a different pattern. An ∼2-Mb region around the origin of replication had an ∼1.2-fold higher coverage than the remaining ∼2.6 Mb centred around the terminus (Figure 3b–d). This ratio of the average coverage between the above-mentioned ∼2-Mb segment around the origin and that for the rest of the chromosome will be referred to as *Rc*. This is not a gradual decrease in coverage from the origin to the terminus, which can be explained by the origin firing multiple times per cell division in rapidly dividing exponential phase cells. Instead, the two domains are separated by sharp boundaries. To further validate the inferred presence of these breakpoints in sequencing coverage, we performed the read coverage distribution analysis for the 16 HS100 colonies that we had sequenced. We noticed the presence of the same pattern, only more prominent than in the population, in three colonies (20% of the colonies; Figure 3e–g). None of these colonies carried a mutation in *rpoS*, but had a mis-sense mutation in the gene *fusA* encoding the translation elongation factor EF-G. This mutation in EF-G appears in a significant proportion of the parental population as well (∼30% of reads) and has expanded in HS100, probably hitch-hiking alongside the structural variation described here. Consistently across the data from the two HS100 populations and the three individual HS100 clones, the two boundaries are located at Insertion Sequence (IS) elements, one boundary being at *insC1* and the other at *insC5. insC1* and *insC5* are identical in sequence and are part of the IS2 transposon, which is present in multiple copies in many *E. coli* strains.

Naively speaking, the data could be explained by both a deletion of the 2.5-Mb region around the terminus (in a sub-population within the colony) or an amplification of the 2-Mb domain around the origin. The former is unlikely, given the presence of the terminus of replication in the region, as well as that of a large number of genes essential for *E. coli* survival. On the other hand, amplification can be promoted by repetitive elements, including rRNA loci and transposable elements such as IS2, by unequal recombination (26). We used computer simulations of read coverage from genomic DNA populations wherein the proposed duplication is prevalent to different extents (Supplementary Figure S8) to systematically show the following. *Rc* values in the 1.2 range, as observed in the read coverage plots for the HS100 population, can be explained by the presence of an amplification in ∼25% of the population, or a deletion in ∼7% of the population. Given these numbers, the chance that three out of 16 sampled clones would display this coverage pattern is three times more likely under the duplication scenario than for the deletion scenario (Supplementary Figure S9). Similarly, *Rc* values in the 1.5–1.8 range—as observed in the single-clone sequencing data—can be explained by an amplification in 60–80% of the genomic DNA molecules (Supplementary Figure S9). That the fold difference does not approach two in these single-clone sequencing data may suggest rapid development of heterogeneity in the population during growth in liquid culture, possibly because of the instability of large duplications (27). We note here that evidence—albeit limited—from the sequencing data does not suggest abnormalities in the copy number of IS2—the repetitive element that forms the boundaries of the duplicated segment—as a result of the duplication: based on the number of IS2 elements encoded in the duplicated and the non-duplicated segments of the chromosome, we expect the copy number of IS2 to be ∼25% higher in the strains with the duplication (assuming *Rc* = 1.8), when compared to the parent. The normalized number of reads that map to these repetitive elements is consistent with this expectation.

### Reversal of the *Δhns-stpA* gene expression imbalance by σ38 inactivation

Next we selected, from the several fully sequenced HS100 clones, one with an inactivating mutation in σ38 *(*referred to as *rpoS^mut^*, carrying a Y61* mutation in addition to deletions of *hns* and *stpA*) and one with the large chromosomal amplification around the origin of replication (*ori^2^*). These clones, alongside a *ΔrpoS-hns-stpA* strain, were subjected to RNA-seq experiments to assess the transcriptional changes occurring in these mutants relative to the parental *Δhns-stpA*. In *rpoS^mut^*, ∼320 genes were upregulated relative to *Δhns-stpA*, and ∼420 downregulated. A large proportion of genes (∼80%; *P* < 10^−10^; Fisher's exact test) differentially expressed in *rpoS^mut^* could be recapitulated in *ΔrpoS-hns-stpA* (Supplementary Figure S10). Further, a significant proportion of genes that were downregulated in *rpoS^mut^* are known targets of σ38 (∼30%; *P* < 10^−10^; Fisher's exact test) identified by microarray experiments from the Hengge lab (28) and/or present in the RegulonDB database (29) (∼400 genes in this collection of σ38 targets). These suggest that the transcriptional effects of *rpoS^mut^* are indeed a result of σ38 inactivation.

**Figure 4.**
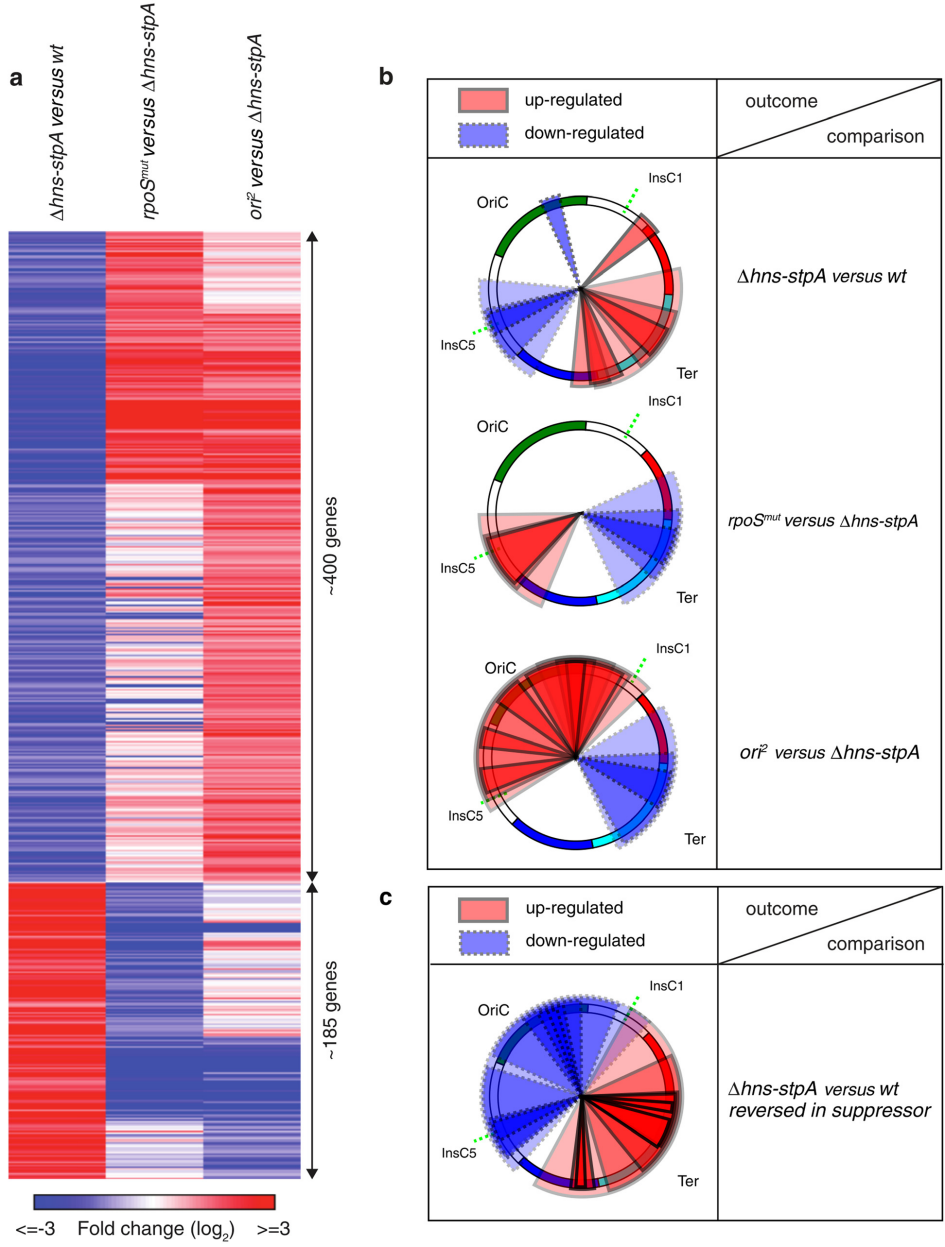
Converging transcriptional outputs of distinct evolutionary strategies. (**a**) Heatmap of differential expression in three comparisons: *Δhns-stpA* versus wild type, rpoS*^mut^* versus *Δhns-stpA* and *ori^2^* versus *Δhns-stpA*. Red indicates upregulation and blue, downregulation. This shows that many of the transcriptional changes observed in the *Δhns-stpA* versus wild-type comparison are reversed in the latter two comparisons. Note that this representation includes only those genes for which differential expression in the *Δhns-stpA* versus wild-type comparison is reversed in at least one of the other two comparisons shown. (**b**) The above result in the context of the chromosome organization, showing that in all the three comparisons presented, up- and downregulation are concentrated in distinct chromosomal regions. In these graphs, each pie slice represents a chromosomal region that is statistically over-represented (*P* < 0.01) for a certain set of genes, defined here by the set of genes differentially expressed (upregulated in red; downregulated in blue) in a stated comparison. To compute whether a set of genes (*G*) is over-represented in a chromosomal window, the method performs randomizations where the gene order is shuffled *in silico*. For each chromosome window, the number of genes from set *G* is calculated from the real genome annotations as well as from the randomized sets. The difference between the two gives a measure of statistical significance. This is calculated for several window sizes. For detailed methods, see Scolari *et al.* (38). The colours in the circle indicate chromosomal macrodomains (green: ori domain; blue: left domain; cyan: ter domain; red: right domain; white: unstructured elements). This representation is derived from the NuST server (http://www.lgm.upmc.fr/nust/). *insC1* and *insC5*, which mark the boundaries of the duplicated domain in *ori^2^*, are marked by the green dashed lines. (**c**) Similar to (b), but shows the subset of gene up- or downregulated in the comparison, but reversed at least in part by one of the two suppressors.

Remarkably, a significant proportion of genes downregulated in *rpoS^mut^* when compared to *Δhns-stpA* had previously been upregulated in *Δhns-stpA* relative to the wild type (∼35%; *P* ∼ 10^−7^; Fisher's exact test; Figure 4a). Similarly, many genes upregulated in the former had been downregulated in the latter comparison (∼50%; *P* < 10^−10^; Fisher's exact test; Figure 4a). We emphasize that upregulation of gene expression resulting from the inactivation of a σ-factor is likely to be an indirect effect. In summary, part of the gene expression state imbalance experienced by *Δhns-stpA* is reversed by a further inactivation of σ38. This spans genes that are upregulated in *Δhns-stpA* as a direct consequence of the loss of H-NS–DNA interactions, as well as those which are downregulated as collateral damage. In contrast, the chance that *rpoS^mut^* would further aggravate the gene expression state of *Δhns-stpA* is significantly less than random (*P* < 10^−5^ for up- and downregulated genes; Fisher's exact test).

Previous research had demonstrated a direct link between H-NS and σ38. In some *Salmonella*, obtaining a *Δhns* mutant is contingent on having a certain *rpoS* background (30). Similarly, in *E. coli* mutations in *rpoS* suppress the growth defect of *hns* mutants (31). In *E. coli*, partial inactivations of H-NS and σ38 interact at a genetic level to produce different physiological outcomes during distinct stages of growth (32). Inactivation of H-NS increases σ38 levels by stabilizing it through a cascade of regulatory interactions (33). This would predict that many σ38 targets should be upregulated when H-NS-mediated gene regulation is impaired, as demonstrated for up to ∼20 genes by the Hengge group (31). However, in our data only a small fraction of genes known to be σ38 targets (∼15% of σ38 targets in our database) are affected by *Δhns-stpA* in comparison to the wild type*.* In fact, this number appears to be slightly less than expected by random chance (*P* < 0.001; Fisher's exact test). Thus, the list of previously known targets of σ38 is substantially exclusive of genes upregulated in *Δhns-stpA* and the effect of rpoS*^mut^* on these targets of H-NS (and StpA).

σ38 is known to activate transcription of several H-NS-repressed genes. This has been shown for selected promoters including those for the stationary phase-related nucleoid-associated protein Dps (34). At present, ∼20 H-NS targets documented in the RegulonDB database are known to be regulated by σ38. Despite this, our data show that the overlap between the targets of H-NS and σ38 is much bigger. What could be the possible sources of this difference? Most σ38 targets known to date were identified by perturbing σ38 activity in a wild-type background. However, there is evidence that H-NS might block access of A+T-rich DNA to other proteins (35), including RNA polymerase (36). Thus, σ38 may have a role in transcribing certain H-NS targets only in the absence of H-NS from these loci. As shown in this study, the list of genes upregulated in *Δhns-stpA* shows a significantly higher overlap with those downregulated in *rpoS^mut^* or *ΔrpoS-hns-stpA* than with those previously known to be regulated by σ38 including those identified in a genome-wide screen (28) (*P* < 10^−10^; Fisher's exact test). Whether these effects of σ38 are direct or not can be further developed by ChIP-chip/ChIP-seq studies of σ38 in a *Δhns-stpA* background.

In summary, σ38 inactivation in a *Δhns-stpA* background results in a partial reversal of the disruption of the gene expression imbalance experienced by *Δhns-stpA*, and the extent to which this occurs may be greater than reported previously.

### Converging transcriptional outputs of the large chromosomal amplification and σ38 inactivation

Towards analysing the interplay between the large chromosomal amplification around the origin of replication and gene expression, we first tested whether genes up- or downregulated in *Δhns-stpA* are differentially distributed between the amplified and the non-amplified segments of the chromosome. Genes that are downregulated in *Δhns-stpA* (relative to the wild type) are slightly more likely to be localized within the amplified segment of the chromosome than those that are upregulated (*P* ∼ 5 × 10^−3^; Fisher's exact test). This is consistent with the observation that H-NS-repressed genes are more likely to be localized around the terminus of replication than the origin (37,38).

The chromosome of the reference *E. coli* used here encodes six repeated IS2 elements (with *insC* homologues), two of which form the boundaries of the amplification. Amplifications promoted by pairs of such elements located around the origin of replication could result in a similar distribution of H-NS-regulated genes between the amplified and the non-amplified part of the chromosome. However, we note that the pair of *insC* elements that we observe in our study is unique in being located symmetrically around the origin of replication (concurrent replication forks), as well as being in or close to the non-structured regions of the chromosome (Figure 3h) (39), which are more likely to be involved in recombination with distal loci. These features might make the reported amplification a more accessible evolutionary strategy than recombination events between other pairs of repeats.

Next, we compared the transcriptome of the evolved *ori^2^* strain against the parental *Δhns-stpA*. For this, to account for possible artefacts that could be introduced in data analysis by the large duplication, we calculated the normalization factor for the RNA-seq data using reads from the non-amplified segment and then used this factor to normalize the whole data set (Supplementary Figures S3 and S4). We find that as many as ∼920 genes are upregulated in *ori^2^* relative to *Δhns-stpA*, whereas ∼210 are downregulated. As expected, a large majority of the upregulated genes (66%; *P* < 10^−10^; Fisher's exact test) are localized to the amplified region of the genome.

Despite the large difference in the number of differentially expressed genes between the *ori^2^* and *rpoS^mut^* evolved strains, they show convergent patterns (Figure 4a). Like the *rpoS^mut^* strain, the *ori^2^* strain also partly reverses the gene expression state disruption in the *Δhns-stpA* mutant. *ori^2^* upregulates the expression levels of a majority of genes (at least 54%; *P* < 10^−10^; Fisher's exact test) that are downregulated in *Δhns-stpA* compared to the wild type. We note that many of these are located in the amplified portion of the chromosome and therefore represent direct effects of the duplication. Interestingly, *ori^2^* also results in the downregulation of several genes upregulated in the *Δhns-stpA* parent: ∼42% (*P* = 4 x 10^−10^; Fisher's exact test) of genes downregulated in *ori^2^* had been upregulated by *Δhns-stpA* relative to the wild type. Overall, a large proportion of genes upregulated in *rpoS^mut^* are also similarly affected in *ori^2^* (62%; *P* < 10^−10^; Fisher's exact test). Despite the similarities between the two evolved strains, the nature of the large amplification in *ori^2^* results in a significantly larger number of genes being upregulated, resulting in a greater reversal of the transcriptional downregulation experienced by *Δhns-stpA* relative to the wild type.

We observe that genes which are upregulated in *ori^2^* show higher wild-type expression levels in mid-exponential phase than those that are not. Consistent with the discussion in the above paragraph, a majority of these genes are downregulated 2-fold or more in *Δhns-stpA* relative to the wild type (Supplementary Figure S11). These lend support to the idea that duplicating a segment of the chromosome around the origin of replication increases the relative dosage and expression of ‘good’ genes with high expression levels during growth and that the expression of these genes is adversely affected in *Δhns-stpA*.

Therefore, inactivation of RpoS and a segmental duplication of ∼40% of the chromosome centred around the origin converge in partially reversing the transcriptional imbalance of the *Δhns-stpA* mutant. It is however not clear whether the suppression of the growth defect of *Δhns-stpA* by these two mutations emerges from the global transcriptional state itself or from their effects on a small subset of genes. For example, H-NS binds to many genes also regulated by the redox global transcription factor FNR (35). We had noted previously that many FNR targets are specifically upregulated in *Δhns-stpA*, but less so in the *Δhns* single mutant (6). Here we observe that amongst the genes upregulated in *Δhns-stpA* and then downregulated by both *rpoS^mut^* and *ori^2^* are several genes involved in anaerobic metabolism under FNR control: these include components of at least two of the three nitrate reductases (Nap and NRA), and formate dehydrogenase. Whether these effects have any direct functional effect on fitness, or are mere coincidences, remains to be addressed. It is a moot point here that both *rpoS^mut^* and *ori^2^* are lost subsequently during the evolution experiment and mutations targeting more local regulatory systems emerge with stronger consequences to fitness. It is curious that amongst these mutations is one in the transcription factor AppY, which is regulated by H-NS and is a horizontally acquired regulator of anaerobiosis.

### Gene expression patterns in the context of chromosomal architecture

**Figure 5.**
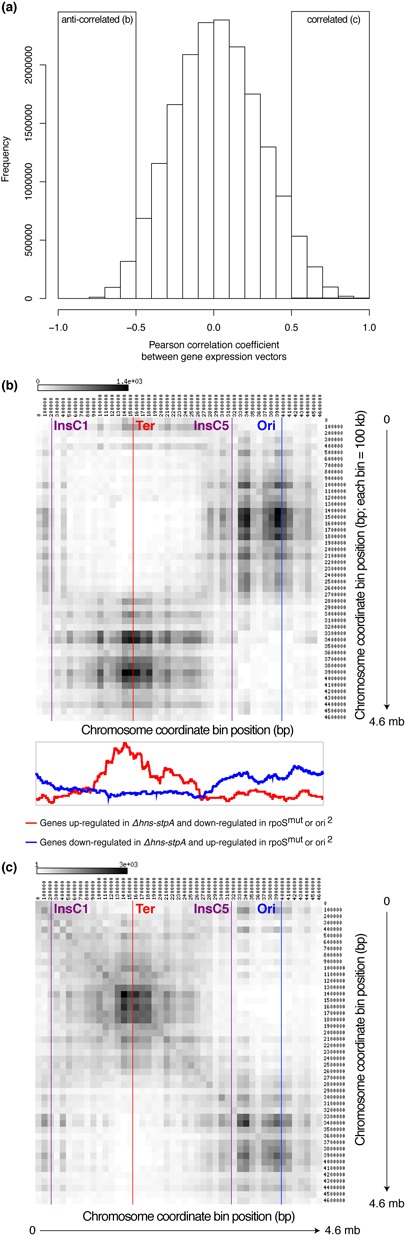
Gene expression correlations in the context of chromosome architecture: this figure summarizes the results of a meta-analysis of 300 publicly-available microarray data. In this analysis, each gene is represented as a vector of ∼300 gene expression values. The Pearson correlation coefficient (PCC) between every pair of these expression vectors is calculated. (**a**) Histogram of the PCC between the expression vectors of all pairs of genes in *E. coli*. A pair of genes was defined as correlated if the PCC between their expression vectors was > = 0.5; and anti-correlated if PCC < = −0.5. As indicated by the histogram, most gene pairs are neither correlated nor anti-correlated. (**b, c**) In these heatmaps, the horizontal and the vertical indicate chromosomal bins of size 100 kb each. The intensity of greyness in each cell represents the number of gene pairs—with (b) anti-correlated or (c) correlated gene expression vectors—encoded in the pair of bins represented by it. White indicates low numbers and black high numbers. These show that there are two large gene expression domains on the *E. coli* chromosome: one domain is centred around the origin of replication and the other around the terminus. Gene pairs within each domain tend to show correlated gene expression patterns across the ∼300 publicly-available microarray experiments, whereas a gene from one domain tends to show an opposing gene expression pattern to one from the other domain. Below (b), numbers of genes differentially expressed across multiple conditions marked in the figure are shown as a function of genomic coordinate, aligned against the positions indicated by the heatmap above. These indicate that the boundaries of the two gene expression domains, obtained from a meta-analysis of ∼300 microarray experiments, are proximal to the transition points (located around the boundaries of the segmental duplication in ori^2^) delineating the opposing gene expression consequences of the mutants investigated in this study. The origin (Ori) and terminus (Ter) of replication, *insC1* and *insC5*, are marked along the horizontal axis in (b) and (c).

We tested for the presence of significant enrichments of differentially expressed genes in specific regions of the chromosome using the NuST web server (39–41). This server divides the chromosome into multiple windows and checks whether a statistically significant number of genes within a given list (for example, genes upregulated in *Δhns-stpA* compared to the wild type) is present in any window, and presents these in a circular chromosome format (Figure 4b). This analysis shows the following: the direct role of H-NS and StpA in silencing gene expression by binding to the chromosome acts around the terminus of replication (37,38). In contrast, the indirect consequence of the *Δhns-stpA* mutant in downregulating many highly expressed genes shows a slight preference to be localized in the origin-half of the chromosome, primarily in the unstructured region located on the left chromosomal arm (referred to as NS-L below). We note here that a part of NS-L is included in the duplicated region in *ori^2^*. In contrast, the (probably) direct effect o*f rpoS^mu^*^t^ is the downregulation of many genes around the terminus; this is consistent with previously published statistical analysis suggesting that stationary phase-induced genes tend to be encoded around the terminus (42). However, this has the indirect consequence of upregulating several genes in NS-L, which had been downregulated by the *Δhns-stpA* mutation. Th*e ori*^2^ mutant, whilst directly upregulating genes in the amplified region around the origin including many within NS-L, also results in the downregulation of several H-NS-regulated genes located around the terminus. Together, upregulation of gene expression in *Δhns-stpA* and its reversal in either of the two suppressors is enriched in the terminus proximal ‘half’ of the chromosome, with the opposing pattern more concentrated around the origin (Figures 4b and 5b, bottom). The limits of the two ‘halves’ of the chromosome are near the boundaries of the segmental duplication in *ori^2^*, marked by *insC1* and *insC5*. These together suggest a three-way link amongst H-NS-dependent gene silencing, σ38 function and chromosome organization i*n E. coli*. These results can be explored further at http://www.lgm.upmc.fr/scolari/srinivasanetal.

Towards investigating the opposing character of gene expression states between the two orthogonal ‘halves’ of the chromosome further, we performed a computational meta-analysis of publicly-available gene expression data across ∼300 different conditions. Note that these conditions go much beyond the limited set of mutants experimentally interrogated in this work, and thus enable derivation of general principles of gene expression patterns in *E. coli*. In this analysis, we represented each gene as a vector of gene expression measures across ∼300 conditions. We then calculated the PCCs (Figure 5a) between the gene expression vectors of all pairs of genes and defined correlated (PCC > = 0.5) and anti-correlated (PCC < = −0.5) gene pairs. In a correlated gene pair, increase (or decrease) in expression of one gene implies a corresponding increase (or decrease) in the expression of the other. On the other hand, increase (or decrease) in expression of a member of an anti-correlated gene pair is associated with a decrease (or increase) in that of the other. In general, most gene pairs are neither correlated nor anti-correlated, i.e. there is no (or at best weak) relationship between the expression vectors of most gene pairs. A small proportion of gene pairs show significant correlation or anti-correlation.

Analysis of anti-correlated gene pairs in the context of their position on the chromosome suggests the presence of two large chromosomal domains: genes localized around the terminus of replication tend to show anti-correlated gene expression vectors to those located on the origin half of the chromosome including the NS-L region (Figure 5b). We observe that the boundaries of these two domains are in proximity to *insC1* and *insC5*, the limits of the segmental duplication in *ori^2^*, which might indicate selection for the positioning of repetitive elements. Therefore, the opposing gene expression pattern between the terminus and the origin of replication, observed in the mutants studied here, appears to be a general trend observed across a large number of genome-wide gene expression studies. This is in contrast to the chromosomal positioning of co-expressed gene pairs, which are more likely to be present in the same half of the chromosome (Figure 5c). However, the mechanisms underlying the persistent opposing gene expression patterns of distinct chromosomal regions—in particular the prospect of chromosome structure influencing these—remain to be addressed.

## CONCLUSION

Gene expression in bacteria is affected by a variety of regulators, including but not limited to nucleoid-associated, chromosome-shaping global transcription factors. Amongst these is the gene silencing system centred around the protein H-NS. H-NS binds to A+T-rich sequence tracts, many of which are horizontally acquired, and keeps them transcriptionally silent (2–4,9,30). We had previously shown, using genome-scale analysis, that the A+T-rich sequences bound by H-NS are intrinsically capable of high gene expression, which, as a cumulative increase in gene expression over ∼20% of the genome, could impose a high metabolic cost on the organism (6). Recent research has further shown that high expression from these loci emerges from pervasive transcription from many promoter-like elements found in A+T-rich sequences (7). We have also shown that the recruitment of a backup system for H-NS-mediated gene silencing—comprising the H-NS-homologue StpA—is more likely to be diverted to genes with high intrinsic transcription (6). Therefore, the gene silencing function of H-NS and its partners is directed towards the silencing of highly transcribable genes. An indirect consequence of the disruption of the H-NS-centred gene silencing mechanism is the downregulation of transcription from a large number (∼15% of all genes) of otherwise highly expressed genes. Thus, de-silencing horizontally acquired genes results in a global disruption of the gene expression state of the cell. How does the cell adapt to such a circumstance, short of re-acquiring the silencing system?

The present work has shown that two distinct evolutionary strategies—multiple mutations each resulting in the inactivation of σ38 (RpoS), the σ-factor for general stress response, as well as a duplication of ∼40% of the genome centred around the origin of replication—converge in partially redressing the transcriptional imbalance of a strain lacking the H-NS/StpA gene silencing system. This suggests a possible role for σ38 in transcription of many H-NS targets. The direct effects of H-NS (37,38) and σ38 (42) regulate gene expression from the terminus half of the chromosome, but result in an indirect and opposing gene expression effect in the non-structured element on the left half of the chromosome (NS-L). The large chromosomal segment that is amplified in *ori^2^* includes part of NS-L. The direct increase in gene expression of the amplified portion of the chromosome results in an indirect decrease in the expression of H-NS/σ38-regulated genes near the terminus. This presents an intriguing connection between the contrasting direct and indirect effects of two distinct global regulatory systems and chromosome architecture. Meta-analysis of microarray data for ∼300 conditions shows the presence of an anti-correlation in expression profiles between genes around the terminus and those around the origin of replication. This analysis indicates that the limits of the segmental duplication described in this study could be the boundary between two opposing gene expression regimes. Given the persistent nature of the opposing gene expression pattern between the origin- and the terminus-half of the chromosome, and the fact that the limits of the two halves are proximal to the duplication-promoting repetitive elements, it is tempting to speculate whether there is selection for the positioning of such repetitive elements relative to the origin of replication. Finally, our study illustrates the value of laboratory evolution experiments, supported by population and single-clone genome and transcriptome sequencing experiments, for studying adaptation of a bacterium to loss of global gene expression control.

The interplay between chromosome architecture and global gene expression (42,43) has been proposed to be an ‘analogue’ layer of regulation laid over the ‘digital’ control imposed by transcription factors such as the Lac repressor (44). A recent paper has shown that the levels of transcription from the well-studied promoter of the *lac* operon are dependent on its position on the genome (45). This interplay between the role of the promoter and that of the larger chromosomal context in gene expression could act at multiple levels, not least replication-dependent copy number gradients (46), DNA supercoiling and the binding of various nucleoid-associated proteins to the DNA, which together impose constraints on the topology of the chromosome (47,48). The exact set of factors that establish the persistent gene expression patterns spanning large tracts of the chromosome needs to be established.

Polymorphisms in *rpoS* are common in both laboratory and environmental/pathogenic isolates of *E. coli* (49). Studies in chemostats have also shown that σ38 inactivation leads to shorter doubling times in low nutrient environments and that such environments select for loss-of-function mutations in *rpoS* (50). It has been argued that the idea of a balance between transcription from the housekeeping σ70 and the stress-response σ38 is best suited for shuttling between feast and famine situations (49). This system does not account for prolonged phase of slow growth, which requires significant expression of housekeeping genes, a situation which might be addressed by an inactivation of σ38. Further, survival in prolonged stationary phase also selects for attenuation of σ38 activity (51).

The emergence of large-scale duplications in response to stress and metabolically limiting conditions is well characterized as a major source of genome plasticity in yeast evolution (27,52–53). The major genetic innovations in bacteria are believed to originate from horizontal gene transfer. However, growth restrictions do result in large segmental duplications in bacteria as well, for example by unequal recombination at repetitive elements such as transposons (26). In many genomes, transient amplifications mediated by repetitive rRNA loci are common (54). Further, duplication of large segments of the chromosome can be selected in low nutrient environments, as shown by seminal studies (55,56). Exposing bacteria to antibiotics targeting DNA replication results in an increase in dosage of genes located close to the origin of replication (57); note however that the scale of this event is significantly smaller than the amplification of ∼40% of the chromosome that we report here. One would expect that amplification of genes around the origin of replication will result in an increase in dosage of many essential and growth-associated genes, whilst decreasing the relative expression of stress-responsive and horizontally acquired genes (46). Though these events are probably transient responses to certain stresses, that large duplications could be stable and define lineages is probably under-appreciated, though indicated by comparative genomics (58). Certain lineages of *Mycobacterium tuberculosis* carry duplications of ∼350 kb (59); this observation of a stable link between a large structural variation and the evolution of *M. tuberculosis* is particularly stark as this is a species that evolves predominantly by point-mutations and deletions. Laboratory evolution (for only 100 generations) of an *E. coli* strain under glucose limitation within chemostats yielded an evolved lineage carrying an ∼180-kb duplication of the 46′ region of the *E. coli* chromosome (60).

The segmental duplication observed here would result in a chromosome with two origins of replication. What are the consequences of such a mutation to DNA replication. Wang *et al.* (61) studied the properties of an artificially constructed *E. coli* cell with two origins of replication, separated from each other by 1 Mb. They observed that the two origins fired synchronously, and replication progressed. Termination of replication from the newly introduced origin did not occur at a diametrically opposite position, but at the wild-type *ter* site. These did not result in any apparent defect in growth or morphology in rich or minimal media, indicating that the bacterium can tolerate multiple origins of replication.

Is there a selective force that underlies selection for RpoS polymorphisms as well as large amplifications? The above-described literature survey suggests that growth restriction under nutrient starvation can select for both RpoS polymorphisms and transient (and occasionally more stable) large duplications. Preliminary experiments performed by us do suggest that the *Δhns-stpA* culture might in fact be experiencing a ‘low-nutrient’ state (Supplementary Figure S12); whether this is the ultimate selective force behind the emergence of the two mutations remains to be tested.

Nevertheless, the brute-force approach of knocking-out the general stress response—thus affecting the expression of a large number of genes—may not necessarily be wise, and large amplifications are unstable. In line with this, these variations exist transiently (27), as we do not see evidence for these in either of the two HS250 populations, or in the four HS250 clones picked for sequencing (Supplementary Figure S2). For example, the HS250 populations harbour mutations that might affect the acid stress response regulon of GadE (62), or the anaerobic-metabolic targets of AppY (63), both of which are influenced by H-NS and StpA. The HS250 clones also carry mutations in RecC, which is involved in recombination, and MutL, which acts in the mismatch repair pathway. These complement the previously reported suppression of the growth defect of *Δhns-stpA* by over-expression of the global transcription factor cAMP Receptor Protein (8). Further examination of the roles, if any, of these mutations in suppressing the growth defect of *Δhns-stpA* is beyond the scope of this work. These specific affects confer a selective advantage over an en-masse inactivation of the σ38 regulon or unstable large amplifications. However, we suggest that the early increase in growth rates, as a result of two easily accessible mutational strategies—the global effects of the large chromosomal amplification (promoted easily by repetitive elements) and σ38 inactivation (a highly mutable locus)—might serve to increase the substrate pool for finer-level selection to act.

## AVAILABILITY

A detailed list of mutations identified in single clones is available in the Supplementary Website http://bugbears.ncbs.res.in/hns_evol and http://www.lgm.upmc.fr/scolari/srinivasanetal.

## ACCESSION NUMBERS

NCBI-SRA SRP043310 and SRP043518.

## SUPPLEMENTARY DATA

Supplementary Data are available at NAR Online.

SUPPLEMENTARY DATA

## References

[1] Madan Babu M., Teichmann S.A., Aravind L. (2006). Evolutionary dynamics of prokaryotic transcriptional regulatory networks. J. Mol. Biol..

[2] Lucchini S., Rowley G., Goldberg M.D., Hurd D., Harrison M., Hinton J.C.D. (2006). H-NS mediates the silencing of laterally acquired genes in bacteria. PLoS Pathog..

[3] Oshima T., Ishikawa S., Kurokawa K., Aiba H., Ogasawara N. (2006). Escherichia coli histone-like protein H-NS preferentially binds to horizontally acquired DNA in association with RNA polymerase. DNA Res..

[4] Dorman C.J. (2007). H-NS, the genome sentinel. Nat. Rev. Microbiol..

[5] Sonden B., Uhlin B.E. (1996). Coordinated and differential expression of histone-like proteins in Escherichia coli: regulation and function of the H-NS analog StpA. EMBO J..

[6] Srinivasan R., Chandraprakash D., Krishnamurthi R., Singh P., Scolari V.F., Krishna S., Seshasayee A.S.N. (2013). Genomic analysis reveals epistatic silencing of ‘expensive’ genes in Escherichia coli K-12. Mol. Biosyst..

[7] Singh S.S., Singh N., Bonocora R.P., Fitzgerald D.M., Wade J.T., Grainger D.C. (2014). Widespread suppression of intragenic transcription initiation by H-NS. Genes Dev..

[8] Johansson J., Balsalobre C., Wang S.Y., Urbonaviciene J., Jin D.J., Sondén B., Uhlin B.E. (2000). Nucleoid proteins stimulate stringently controlled bacterial promoters: a link between the cAMP-CRP and the (p)ppGpp regulons in Escherichia coli. Cell.

[9] Kahramanoglou C., Seshasayee A.S.N., Prieto A.I., Ibberson D., Schmidt S., Zimmermann J., Benes V., Fraser G.M., Luscombe N.M. (2011). Direct and indirect effects of H-NS and Fis on global gene expression control in Escherichia coli. Nucleic Acids Res..

[10] Doyle M., Fookes M., Ivens A., Mangan M.W., Wain J., Dorman C.J. (2007). An H-NS-like stealth protein aids horizontal DNA transmission in bacteria. Science.

[11] Datsenko K.A., Wanner B.L. (2000). One-step inactivation of chromosomal genes in Escherichia coli K-12 using PCR products. Proc. Natl Acad. Sci. U.S.A..

[12] Li H., Durbin R. (2010). Fast and accurate long-read alignment with Burrows-Wheeler transform. Bioinformatics.

[13] Li H., Handsaker B., Wysoker A., Fennell T., Ruan J., Homer N., Marth G., Abecasis G., Durbin R., 1000 Genome Project Data Processing Subgroup (2009). The Sequence Alignment/Map format and SAMtools. Bioinformatics.

[14] Koboldt D.C., Chen K., Wylie T., Larson D.E., McLellan M.D., Mardis E.R., Weinstock G.M., Wilson R.K., Ding L. (2009). VarScan: variant detection in massively parallel sequencing of individual and pooled samples. Bioinformatics.

[15] Deatherage D.E., Barrick J.E. (2014). Identification of mutations in laboratory-evolved microbes from next-generation sequencing data using breseq. Methods Mol. Biol..

[16] Langmead B., Trapnell C., Pop M., Salzberg S.L. (2009). Ultrafast and memory-efficient alignment of short DNA sequences to the human genome. Genome Biol..

[17] Keseler I.M., Bonavides-Martínez C., Collado-Vides J., Gama-Castro S., Gunsalus R.P., Johnson D.A., Krummenacker M., Nolan L.M., Paley S., Paulsen I.T. (2009). EcoCyc: a comprehensive view of Escherichia coli biology. Nucleic Acids Res..

[18] Robinson M.D., McCarthy D.J., Smyth G.K. (2010). edgeR: a Bioconductor package for differential expression analysis of digital gene expression data. Bioinformatics.

[19] Smyth G.K. (2004). Linear models and empirical bayes methods for assessing differential expression in microarray experiments. Stat. Appl. Genet. Mol. Biol..

[20] Faith J.J., Driscoll M.E., Fusaro V.A., Cosgrove E.J., Hayete B., Juhn F.S., Schneider S.J., Gardner T.S. (2008). Many Microbe Microarrays Database: uniformly normalized Affymetrix compendia with structured experimental metadata. Nucleic Acids Res..

[21] Chandraprakash D., Seshasayee A.S.N. (2014). Inhibition of factor-dependent transcription termination in Escherichia coli might relieve xenogene silencing by abrogating H-NS-DNA interactions in vivo. J. Biosci..

[22] Browning D.F., Busby S.J. (2004). The regulation of bacterial transcription initiation. Nat. Rev. Microbiol..

[23] Zaslaver A., Kaplan S., Bren A., Jinich A., Mayo A., Dekel E., Alon U., Itzkovitz S. (2009). Invariant distribution of promoter activities in Escherichia coli. PLoS Comput. Biol..

[24] Chubiz L.M., Lee M.-C., Delaney N.F., Marx C.J. (2012). FREQ-Seq: a rapid, cost-effective, sequencing-based method to determine allele frequencies directly from mixed populations. PloS One.

[25] Hong S.H., Wang X., Wood T.K. (2010). Controlling biofilm formation, prophage excision and cell death by rewiring global regulator H-NS of Escherichia coli. Microb. Biotechnol..

[26] Andersson D.I., Hughes D. (2009). Gene amplification and adaptive evolution in bacteria. Annu. Rev. Genet..

[27] Yona A.H., Manor Y.S., Herbst R.H., Romano G.H., Mitchell A., Kupiec M., Pilpel Y., Dahan O. (2012). Chromosomal duplication is a transient evolutionary solution to stress. Proc. Natl Acad. Sci. U.S.A..

[28] Weber H., Polen T., Heuveling J., Wendisch V.F., Hengge R. (2005). Genome-wide analysis of the general stress response network in Escherichia coli: sigmaS-dependent genes, promoters, and sigma factor selectivity. J. Bacteriol..

[29] Gama-Castro S., Jiménez-Jacinto V., Peralta-Gil M., Santos-Zavaleta A., Peñaloza-Spinola M.I., Contreras-Moreira B., Segura-Salazar J., Muñiz-Rascado L., Martínez-Flores I., Salgado H. (2008). RegulonDB (version 6.0): gene regulation model of Escherichia coli K-12 beyond transcription, active (experimental) annotated promoters and Textpresso navigation. Nucleic Acids Res..

[30] Navarre W.W., Porwollik S., Wang Y., McClelland M., Rosen H., Libby S.J., Fang F.C. (2006). Selective silencing of foreign DNA with low GC content by the H-NS protein in Salmonella. Science.

[31] Barth M., Marschall C., Muffler A., Fischer D., Hengge-Aronis R. (1995). Role for the histone-like protein H-NS in growth phase-dependent and osmotic regulation of sigma S and many sigma S-dependent genes in Escherichia coli. J. Bacteriol..

[32] Chib S., Mahadevan S. (2012). Involvement of the global regulator H-NS in the survival of Escherichia coli in stationary phase. J. Bacteriol..

[33] Battesti A., Tsegaye Y.M., Packer D.G., Majdalani N., Gottesman S. (2012). H-NS regulation of IraD and IraM antiadaptors for control of RpoS degradation. J. Bacteriol..

[34] Grainger D.C., Goldberg M.D., Lee D.J., Busby S.J.W. (2008). Selective repression by Fis and H-NS at the Escherichia coli dps promoter. Mol. Microbiol..

[35] Myers K.S., Yan H., Ong I.M., Chung D., Liang K., Tran F., Keleş S., Landick R., Kiley P.J. (2013). Genome-scale analysis of Escherichia coli FNR reveals complex features of transcription factor binding. PLoS Genet..

[36] Singh S.S., Grainger D.C. (2013). H-NS can facilitate specific DNA-binding by RNA polymerase in AT-rich gene regulatory regions. PLoS Genet..

[37] Zarei M., Sclavi B., Cosentino Lagomarsino M. (2013). Gene silencing and large-scale domain structure of the E. coli genome. Mol. Biosyst..

[38] Scolari V.F., Bassetti B., Sclavi B., Lagomarsino M.C. (2011). Gene clusters reflecting macrodomain structure respond to nucleoid perturbations. Mol. Biosyst..

[39] Valens M., Penaud S., Rossignol M., Cornet F., Boccard F. (2004). Macrodomain organization of the Escherichia coli chromosome. EMBO J..

[40] Scolari V.F., Zarei M., Osella M., Lagomarsino M.C. (2012). NuST: analysis of the interplay between nucleoid organization and gene expression. Bioinformatics.

[41] Mathelier A., Carbone A. (2010). Chromosomal periodicity and positional networks of genes in Escherichia coli. Mol. Syst. Biol..

[42] Sobetzko P., Travers A., Muskhelishvili G. (2012). Gene order and chromosome dynamics coordinate spatiotemporal gene expression during the bacterial growth cycle. Proc. Natl Acad. Sci. U.S.A..

[43] Dorman C.J. (2013). Genome architecture and global gene regulation in bacteria: making progress towards a unified model?. Nat. Rev. Microbiol..

[44] Marr C., Geertz M., Hütt M.-T., Muskhelishvili G. (2008). Dissecting the logical types of network control in gene expression profiles. BMC Syst. Biol..

[45] Bryant J.A., Sellars L.E., Busby S.J.W., Lee D.J. Chromosome position effects on gene expression in Escherichia coli K-12. Nucleic Acids Res..

[46] Rocha E.P.C. (2004). The replication-related organization of bacterial genomes. Microbiology.

[47] Le T.B.K., Imakaev M.V., Mirny L.A., Laub M.T. (2013). High-resolution mapping of the spatial organization of a bacterial chromosome. Science.

[48] Umbarger M.A., Toro E., Wright M.A., Porreca G.J., Baù D., Hong S.-H., Fero M.J., Zhu L.J., Marti-Renom M.A., McAdams H.H. (2011). The three-dimensional architecture of a bacterial genome and its alteration by genetic perturbation. Mol. Cell.

[49] Ferenci T. (2003). What is driving the acquisition of mutS and rpoS polymorphisms in Escherichia coli?. Trends Microbiol..

[50] Chen G., Patten C.L., Schellhorn H.E. (2004). Positive selection for loss of RpoS function in Escherichia coli. Mutat. Res..

[51] Zambrano M.M., Siegele D.A., Almirón M., Tormo A., Kolter R. (1993). Microbial competition: Escherichia coli mutants that take over stationary phase cultures. Science.

[52] Payen C., Di Rienzi S.C., Ong G.T., Pogachar J.L., Sanchez J.C., Sunshine A.B., Raghuraman M.K., Brewer B.J., Dunham M.J. (2014). The dynamics of diverse segmental amplifications in populations of Saccharomyces cerevisiae adapting to strong selection. G3.

[53] Koszul R., Fischer G. (2009). A prominent role for segmental duplications in modeling eukaryotic genomes. C. R. Biol..

[54] Anderson P., Roth J. (1981). Spontaneous tandem genetic duplications in Salmonella typhimurium arise by unequal recombination between rRNA (rrn) cistrons. Proc. Natl Acad. Sci. U.S.A..

[55] Sonti R.V., Roth J.R. (1989). Role of gene duplications in the adaptation of Salmonella typhimurium to growth on limiting carbon sources. Genetics.

[56] Straus D.S. (1975). Selection for a large genetic duplication in Salmonella typhimurium. Genetics.

[57] Slager J., Kjos M., Attaiech L., Veening J.-W. (2014). Antibiotic-induced replication stress triggers bacterial competence by increasing gene dosage near the origin. Cell.

[58] Kong S.-G., Fan W.-L., Chen H.-D., Hsu Z.-T., Zhou N., Zheng B., Lee H.-C. (2009). Inverse symmetry in complete genomes and whole-genome inverse duplication. PloS One.

[59] Domenech P., Kolly G.S., Leon-Solis L., Fallow A., Reed M.B. (2010). Massive gene duplication event among clinical isolates of the Mycobacterium tuberculosis W/Beijing family. J. Bacteriol..

[60] Maharjan R.P., Gaffé J., Plucain J., Schliep M., Wang L., Feng L., Tenaillon O., Ferenci T., Schneider D. (2013). A case of adaptation through a mutation in a tandem duplication during experimental evolution in Escherichia coli. BMC Genomics.

[61] Wang X., Lesterlin C., Reyes-Lamothe R., Ball G., Sherratt D.J. (2011). Replication and segregation of an Escherichia coli chromosome with two replication origins. Proc. Natl Acad. Sci. U.S.A..

[62] Hommais F., Krin E., Coppée J.-Y., Lacroix C., Yeramian E., Danchin A., Bertin P. (2004). GadE (YhiE): a novel activator involved in the response to acid environment in Escherichia coli. Microbiology.

[63] Atlung T., Brøndsted L. (1994). Role of the transcriptional activator AppY in regulation of the cyx appA operon of Escherichia coli by anaerobiosis, phosphate starvation, and growth phase. J. Bacteriol..

