# Supplementary material for: The genome-scale interplay amongst xenogene silencing, stress response and chromosome architecture in Escherichia coli
Source: Nucleic Acids Res. 2014 Nov 27;43(1):295–308. doi: 10.1093/nar/gku1229 (PMC4288151; doi:10.1093/nar/gku1229)
Supplement: SUPPLEMENTARY DATA [file supp_43_1_295__index.html]

The genome-scale interplay amongst xenogene silencing, stress response and chromosome architecture in Escherichia coli — SUPPLEMENTARY DATA 

# The genome-scale interplay amongst xenogene silencing, stress response and chromosome architecture in *Escherichia coli*

## SUPPLEMENTARY DATA

**Files in this Data Supplement:**

- SUPPLEMENTARY DATA
- SUPPLEMENTARY DATA
