# Supplementary material for: The genome-scale interplay amongst xenogene silencing, stress response and chromosome architecture in Escherichia coli
Source: Nucleic Acids Res. 2014 Nov 27;43(1):295–308. doi: 10.1093/nar/gku1229 (PMC4288151; doi:10.1093/nar/gku1229)
Supplement: SUPPLEMENTARY DATA [file supp_gku1229_nar-02409-z-2014-File007.pdf]

The genome-scale interplay among xenogene silencing, stress response and chromosome architecture in E. coli

Rajalakshmi Srinivasan[1,2], Vittore Ferdinando Scolari[1,2,3], Marco Cosentino Lagomarsino[3], Aswin Sai Narain Seshasayee[1],*

1. National Centre for Biological Sciences, GKVK, Bellary Road, Bangalore 560065, India

2. Manipal University, Manipal 576104, India

3. Genomic Physics Group, UMR 7238 CNRS Microorganism Genomics, UPMC, Paris, France

*For correspondence: aswin@ncbs.res.in

Supplementary Material

Supplementary Figure 1

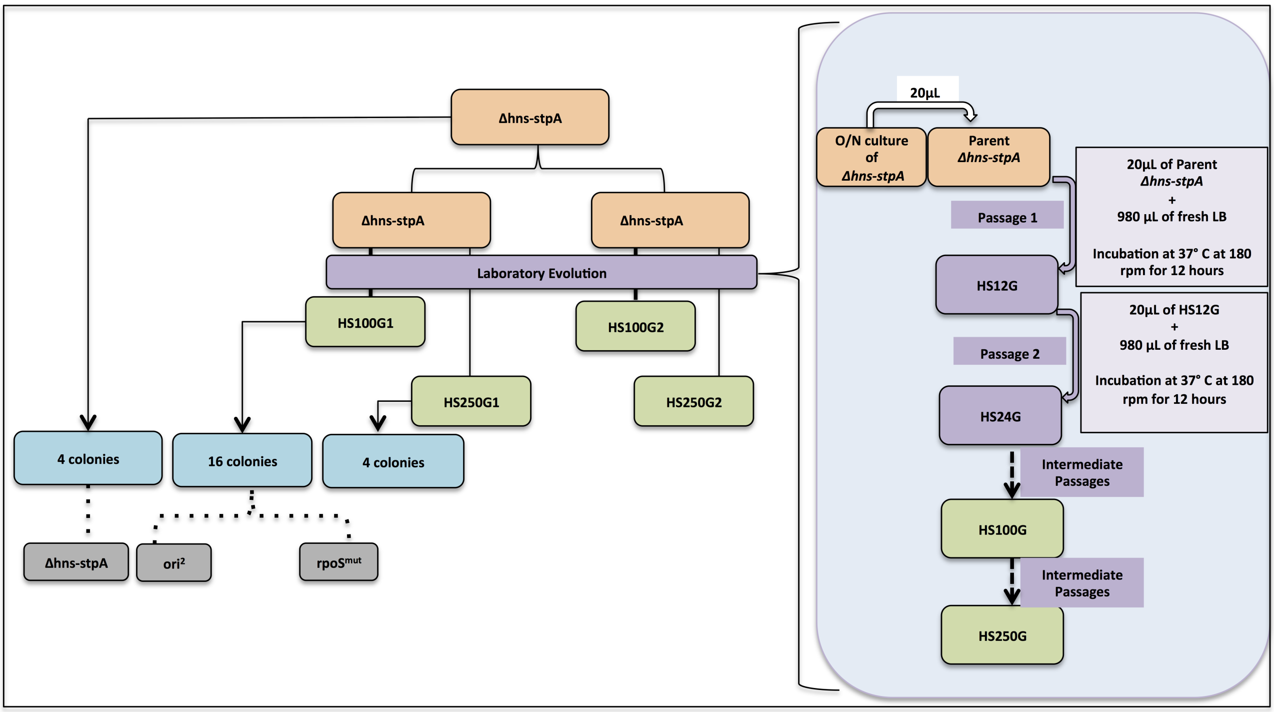

The above figure shows a schematic representation of our laboratory evolution experiment. Green coloured boxes represent samples taken for population genomic DNA sequencing; cyan boxes show clones isolated for single colony paired-end genomic DNA sequencing; strains selected for transcriptome experiments are in grey. On the right, a representation of the laboratory evolution experimental approach is shown.

Supplementary Figure 2

For a thorough description of mutations please visit http://bugbears.ncbs.res.in/hns_evol.

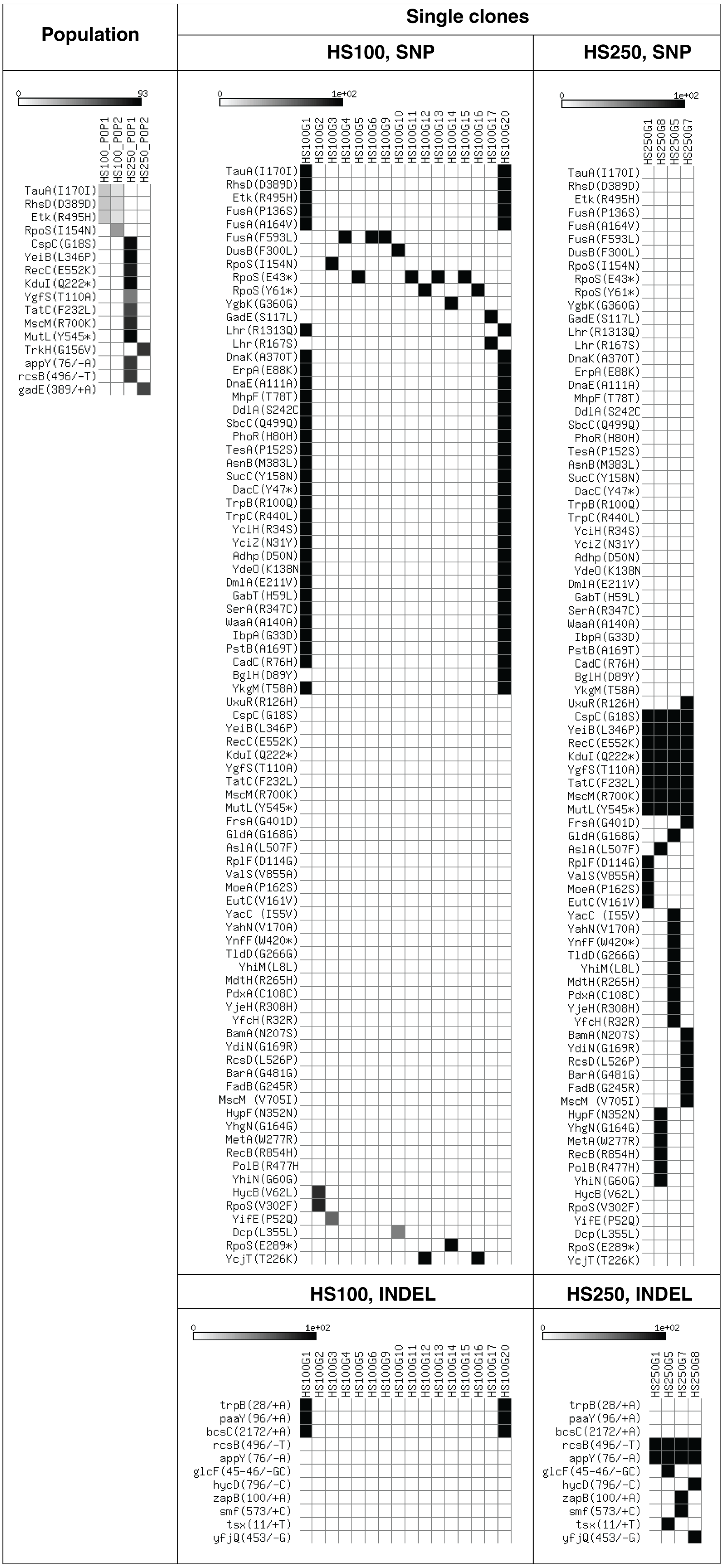
Population
Single clones
HS100, SNP
HS250, SNP
HS100, INDEL
HS250, INDEL
0
93
1e+02
HS100_POP1 HS100_POP2 HS250_POP1 HS250_POP2
TauA(I170I) RhsD(D389D) Etk(R495H) RpoS(I154N) CspC(G18S) YeiB(L346P) RecC(E552K) KduI(Q222*) YgfS(T110A) TatC(F232L) MscM(R700K) MutL(Y545*) TrkH(G156V) appY(76/-A) rcsB(496/-T) gadE(389/+A)
HS100G1 HS100G2 HS100G3 HS100G4 HS100G5 HS100G6 HS100G9 HS100G10 HS100G11 HS100G12 HS100G13 HS100G14 HS100G15 HS100G16 HS100G17 HS100G20
HS250G1 HS250G8 HS250G5 HS250G7
TauA(I170I) RhsD(D389D) Etk(R495H) FusA(P136S) FusA(A164V) FusA(F593L) DusB(F300L) RpoS(I154N) RpoS(E43*) RpoS(Y61*) YgbK(G360G) GadE(S117L) Lhr(R1313Q) Lhr(R167S) DnaK(A370T) ErpA(E88K) DnaE(A111A) MhpF(T78T) DdlA(S242C SbcC(Q499Q) PhoR(H80H) TesA(P152S) AsnB(M383L) SucC(Y158N) DacC(Y47*) TrpB(R100Q) TrpC(R440L) YciH(R34S) YciZ(N31Y) Adhp(D50N) YdeO(K138N DmlA(E211V) GabT(H59L) SerA(R347C) WaaA(A140A) IbpA(G33D) PstB(A169T) CadC(R76H) BglH(D89Y) YkgM(T58A) UxuR(R126H) CspC(G18S) YeiB(L346P) RecC(E552K) KduI(Q222*) YgfS(T110A) TatC(F232L) MscM(R700K) MutL(Y545*) FrsA(G401D) GldA(G168G) AslA(L507F) RplF(D114G) ValS(V855A) MoeA(P162S) EutC(V161V) YacC (I55V) YahN(V170A) YnfF(W420*) TldD(G266G) YhiM(L8L) MdtH(R265H) PdxA(C108C) YjeH(R308H) YfcH(R32R) BamA(N207S) YdiN(G169R) RcsD(L526P) BarA(G481G) FadB(G245R) MscM (V705I) HypF(N352N) YhgN(G164G) MetA(W277R) RecB(R854H) PolB(R477H YhiN(G60G) HycB(V62L) RpoS(V302F) YifE(P52Q) Dcp(L355L) RpoS(E289*) YcjT(T226K)
HS250G1 HS250G5 HS250G7 HS250G8
trpB(28/+A) paaY(96/+A) bcsC(2172/+A) rcsB(496/-T) appY(76/-A) glcF(45-46/-GC) hycD(796/-C) zapB(100/+A) smf(573/+C) tsx(11/+T) yfjQ(453/-G)

The figure shows a heatmap of single nucleotide substitutions, and lists of genes with indels, found in the genomes of populations of evolved bacteria (left panel) and single clones (middle panel for HS100 and right panel for HS250) obtained from them. For SNPs, the amino acid position that is mutated is mentioned; for indels, the variant base position within the nucleotide sequence is marked. The colour in each cell indicates the % of sequence reads that support the mutation (white ~ 0; black ~100%). For all these analysis NC_000913.2 version of the *E. coli* genome was used. The more recent NC_000913.3 version of the genome is slightly longer because of the presence of an insertion element, which is not present in the version of the bacterium we use.

In the population sequencing data, only mutations represented by more than 20% of sequencing reads, and not found in any read from the parental population are shown. Note that this heatmap is indicative of the heterogeneity in the evolved population, at least in HS100; the numbers of mutations would differ with changes in the cutoffs used.

The clones are represented by the nomenclature HS100G<N> or HS250G<N> , where N is a number internally assigned to a colony. HS100G4 is *ori*^{2} and HS100G12 is *rpoS*^{mut}. Other clones carrying the segmental duplication are HS100G6 and HS100G9. This is aimed at providing a birds-eye view of the variations seen in our experiment. Mutations called by the BRESEQ pipeline can be thoroughly investigated at http://bugbears.ncbs.res.in/hns_evol.

Supplementary Figure 3

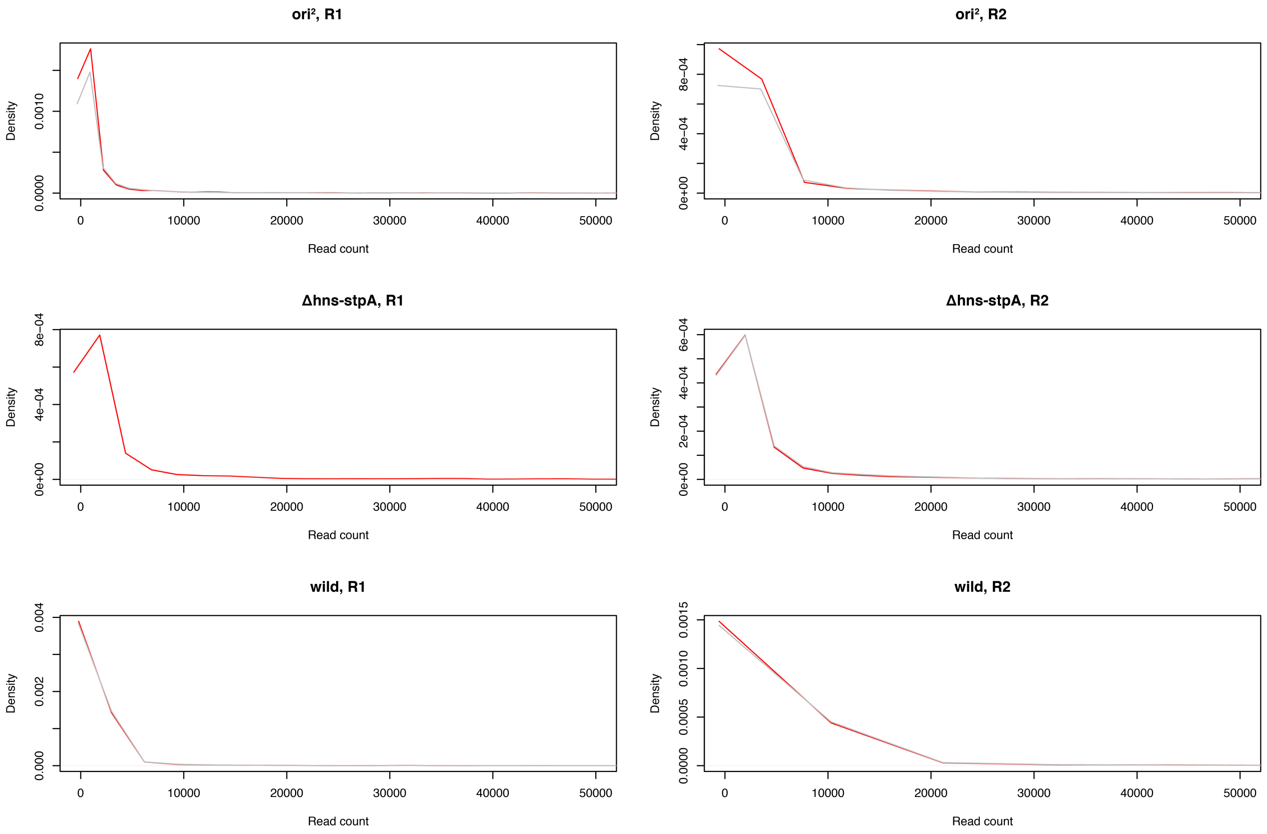

The figure shows read count distributions for all genes (grey lines; overlapping with red lines at times), and of those genes located in the non-amplified portion (red) of the chromosome. The strain is named above the plot; R1 and R2 indicate the two biological replicates. This shows that the read count distribution is different between the amplified and the non-amplified segments of the genome only in ori^2.

Supplementary Figure 4

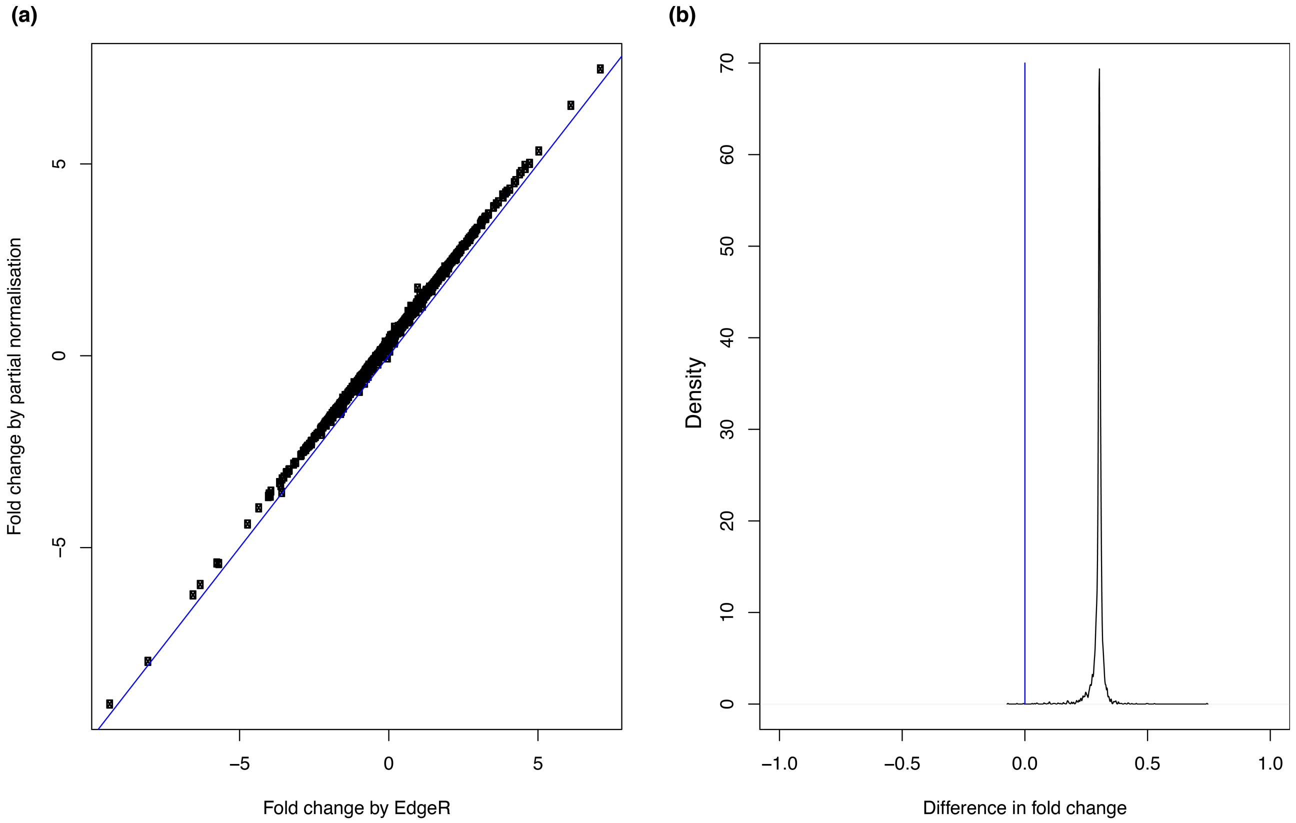

The figure shows a (a) scatter plot indicating the correlation between the fold changes (*ori*^{2} minus Δ*hns-stpA*) between our normalisation method followed by LIMMA differential expression calculation (called "*partial normalisation*" procedure) and the standard EdgeR procedure, with the 45^0 line drawn in blue; (b) a distribution of difference in fold changes between the two procedures (partial normalisation minus EdgeR). All fold change measures are on the log, base 2 scale.

Supplementary Figure 5

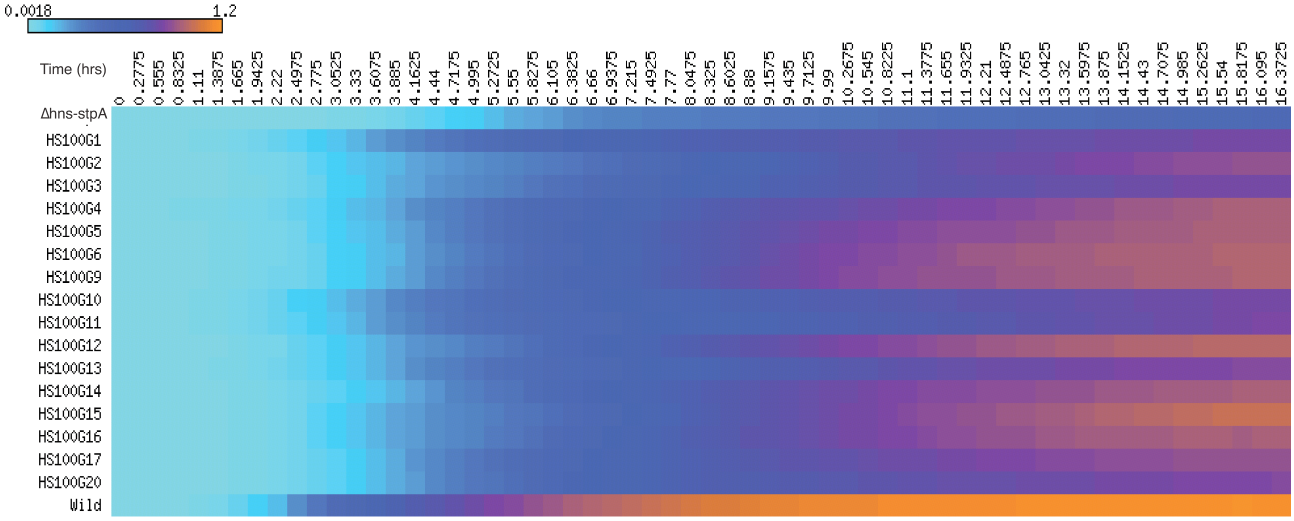

This figure shows a heatmap representation of growth curves for each of the 16 clones from the HS100 population picked for whole-genome sequencing. Each row represents a clone, and each column a time point during a single batch culture. The clones are represented by the nomenclature HS100G<N>, where N is a number internally assigned to a colony. HS100G4 is *ori*^{2} and HS100G12 is *rpoS*^{mut}. HS100G6 and HS100G9 carry the segmental duplication. The colour in each cell is indicative of OD_{60}, with cyan representing lowest values and orange the highest.

Supplementary Figure 6

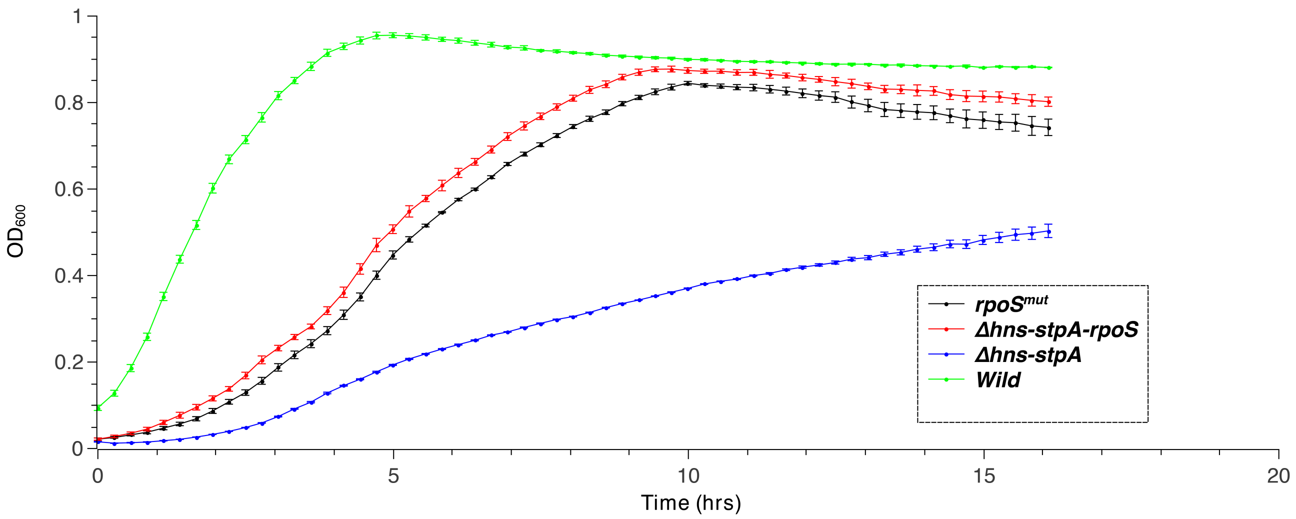

This figure shows growth curves of *E. coli* Δ*hns-stpA* (blue), and *rpoS*^{mut} (black) and Δ*hns-stpA-rpoS* (red). Also shown is the growth curve of the wildtype *E. coli* K12 MG1655 (green). All these growth experiments were performed in a multi-well plate format. The error bars represent the standard error across 2 biological and 6 technical replicates.

Supplementary Figure 7

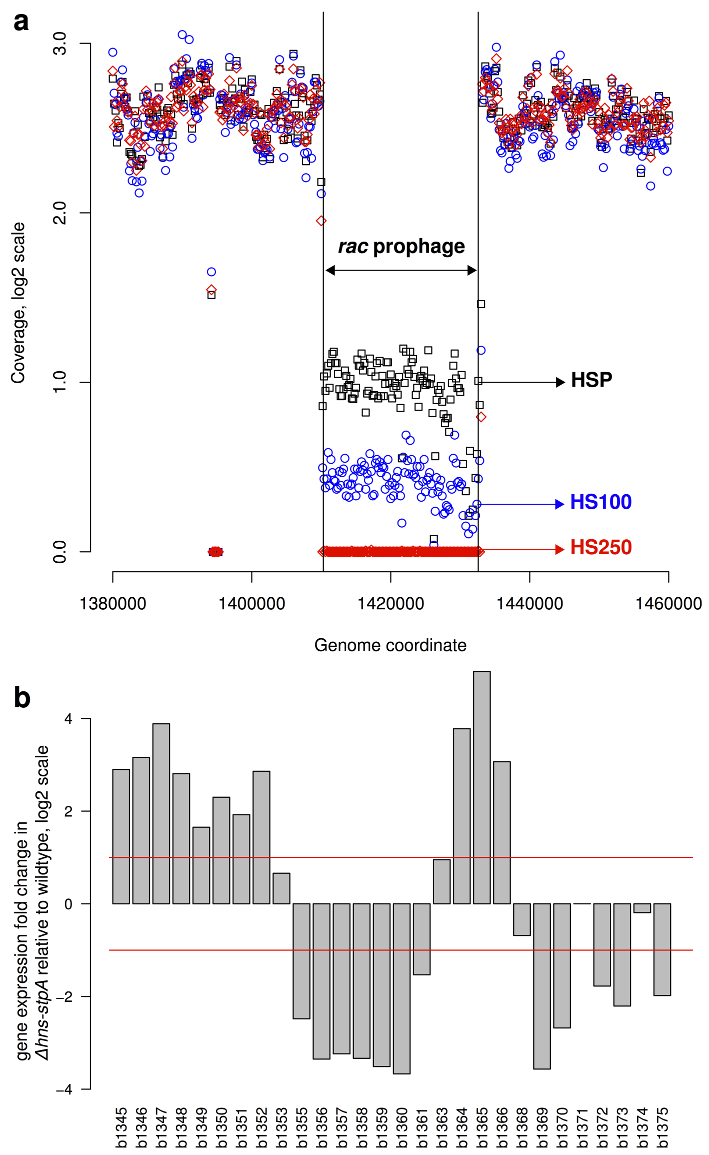

Analysis of the read coverage distribution of the parental Δ*hns-stpA* genome (HSP) revealed a deletion of the *rac* prophage, presumably in a sub-population. We suspect that a proportion of cells loses the *rac* prophage when the glycerol stock of a single Δ*hns-stpA* colony is streaked on an LB-agar plate, and during the subsequent batch liquid culture used for nucleic acid extraction. At this stage, the *rac* prophage is not fully lost from the population yet: many reads do map to the prophage, with an ~3-fold difference in coverage between the *rac* locus and the flanking regions (Panel A above, which shows the read coverage of the rac prophage and flanking regions as a function of genome coordinate; black – parent, blue – HS100 population, red – HS250 population). That the *rac* prophage is not deleted completely from the parental population is further supported by our previously-reported transcriptome of Δ*hns-stpA*, which showed up-regulation of certain *rac* prophage genes, including the gene for the toxin *kilR*, in the mutant when compared with the wildtype (Panel B above). Nevertheless, *rac* excision is presumably very common, as we noticed its deletion

in the genome sequences of each of four single Δ*hns-stpA* colonies from the streaked plate. This is consistent with a previous study (Hong et al. Microbial Biotechnology. 3: 344-356. 2010), which had reported rapid excision of *rac* in Δ*hns* and a H-NSK57N mutant where the oligomerisation of the protein was disrupted. In the HS100 populations, fewer reads map to the *rac* region, whereas hardly any read aligns to this locus in the HS250 populations (Panel A).

Supplementary Figure 8

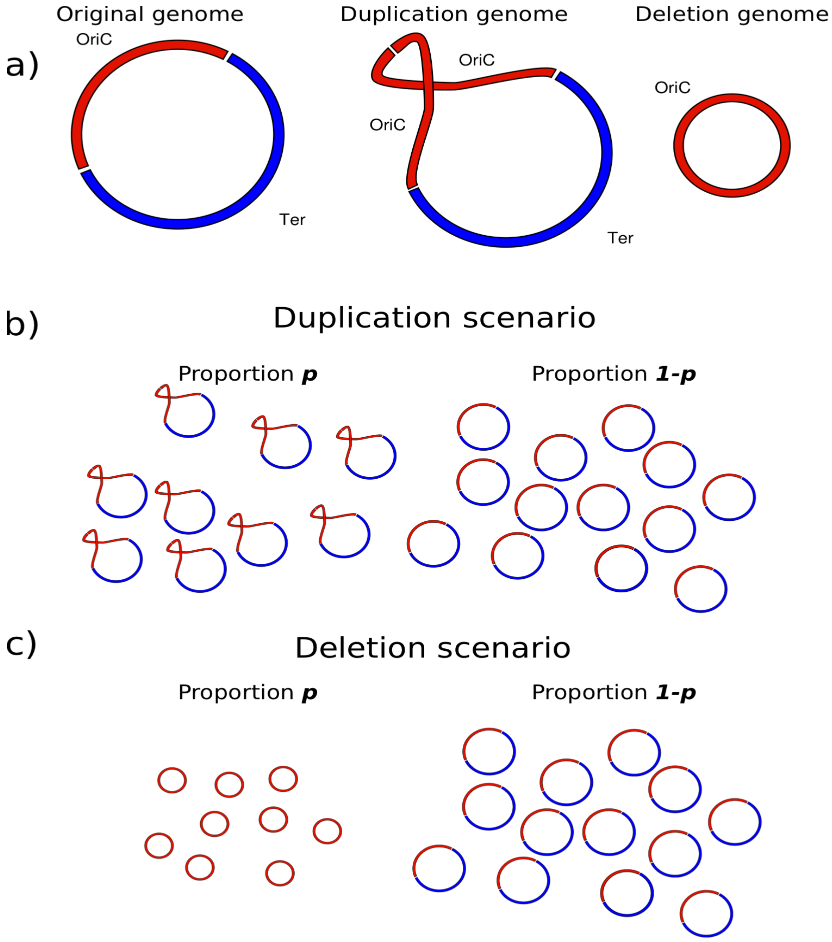

From the sequencing data for HS100, we infer the presence of an amplification of ~40% of the genome, centred around the origin of replication. In theory, there is a remote possibility that this could arise from a deletion of the remaining ~60% of the genome, around the terminus of replication (panel **a** in the figure above). This might be possible in cells with multiple nucleoids, with a small proportion of the genomic DNA molecules carrying such a deletion. Using the simulation approach described below, we estimated the proportion of nucleoids carrying an amplification or a deletion, for a given difference in coverage between the Ori-centred and the Ter-centred segments of the chromosomes.

What we describe below is a computer experiment simulating the expected experimental outcome of sequencing a certain population of genomic DNA molecules. This allows us to impose certain conditions (example: proportion of genomic DNA molecules with a duplication or a deletion), and build expectations of quantitative measures resulting from the sequencing of the population of DNA. These expected measures can then be compared to experimental data to derive conclusions.

We simulated random fragmentation of the chromosome under the two above-described scenarios, as follows (panel **b** and **c** in the figure): a portion p of the genomic DNA molecules either underwent a global duplication of the High Coverage region (duplication scenario) or underwent a deletion of the Low

Coverage region (deletion scenario). For example $p = 0.1$ in the duplication scenario implies that 90% of the genomic DNA molecules are wildtype and the remaining with the duplication.

Both scenarios – deletion and duplication - would carry an advantage in selection in the sense that they would relatively increase the expression of growth-associated genes in the Ori region in comparison to the Ter region. The first scenario does so due to the higher copy number of the duplicated region but it comes with a trade-off of reduced fitness because the chromosome replication time depends linearly on its length. In the second scenario, the effect on gene expression is mediated by the reduction in copy numbers of the genes belonging to the deleted region, which encodes many horizontally acquired genes including eight of the nine cryptic prophages; these cells suffer from the deletion of multiple essential genes belonging to the Ter region, and can survive possibly only by polyploidy.

The simulation first generates 8000000 random fragments from the population of genomic DNA molcules described by the two scenarios. This is similar to the fragmentation of the genomic DNA molecule that is performed before sequencing. For each fragment, the sequences of 100-bases on either end are written down into the simulated sequencing data file – akin to the paired-end sequencing technique. These data as processed in a manner similar to real sequencing data, except for quality controls as the simulated data assumes 100% accuracy. The sequencing coverage is plotted against chromosomal coordinates, for various scenarios (varying p for duplication and deletion), and is shown in the figure below (panel a). The fold-change in the coverage between the high and the low coverage regions is predicted to depend only on p and on the length fraction of the higher coverage region over the whole genome δ in the two scenarios considered. The results of the simulations lead to the interpolation formulas described in the figure below (panel b).

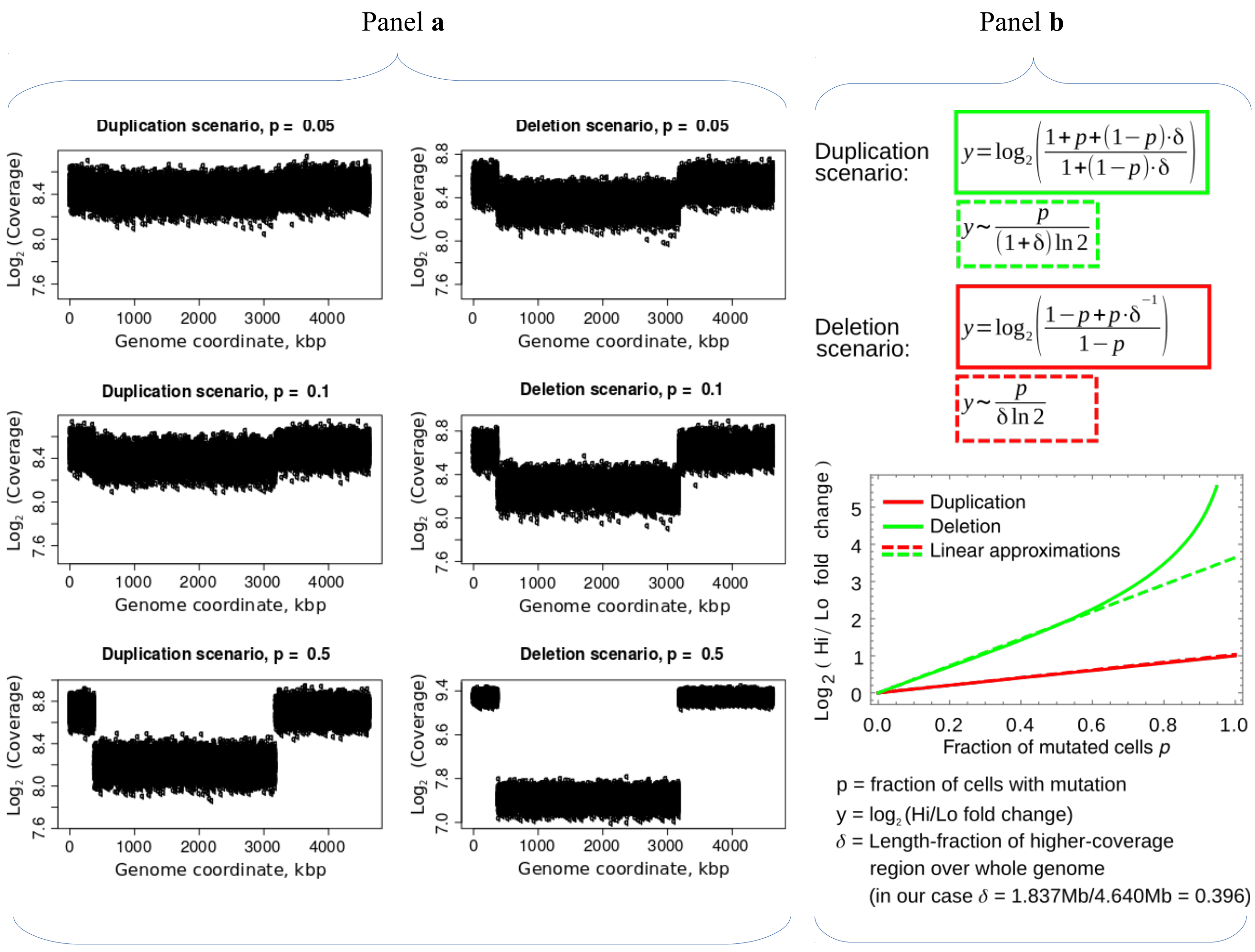

In the above figure, panel **a** shows simulated read coverage coverage as a function of genome coordinate for different values of p in the deletion and the duplication scenarios. After generating 8000000 of random fragments for a given population, we plot the histogram of the fragments along the genome after binning it into segments each 200 base pair long. The data is plotted for $p = 0.05, 0.1, 0.5$ in the two different scenarios. Panel **b** illustrates the interpolation formulae for coverage fold-change as a function of p and δ. These formulae are useful to estimate the value of p from coverage data; an expression is given for the complete relation as well as for the first-order linear approximation in p.

Supplementary Figure 9

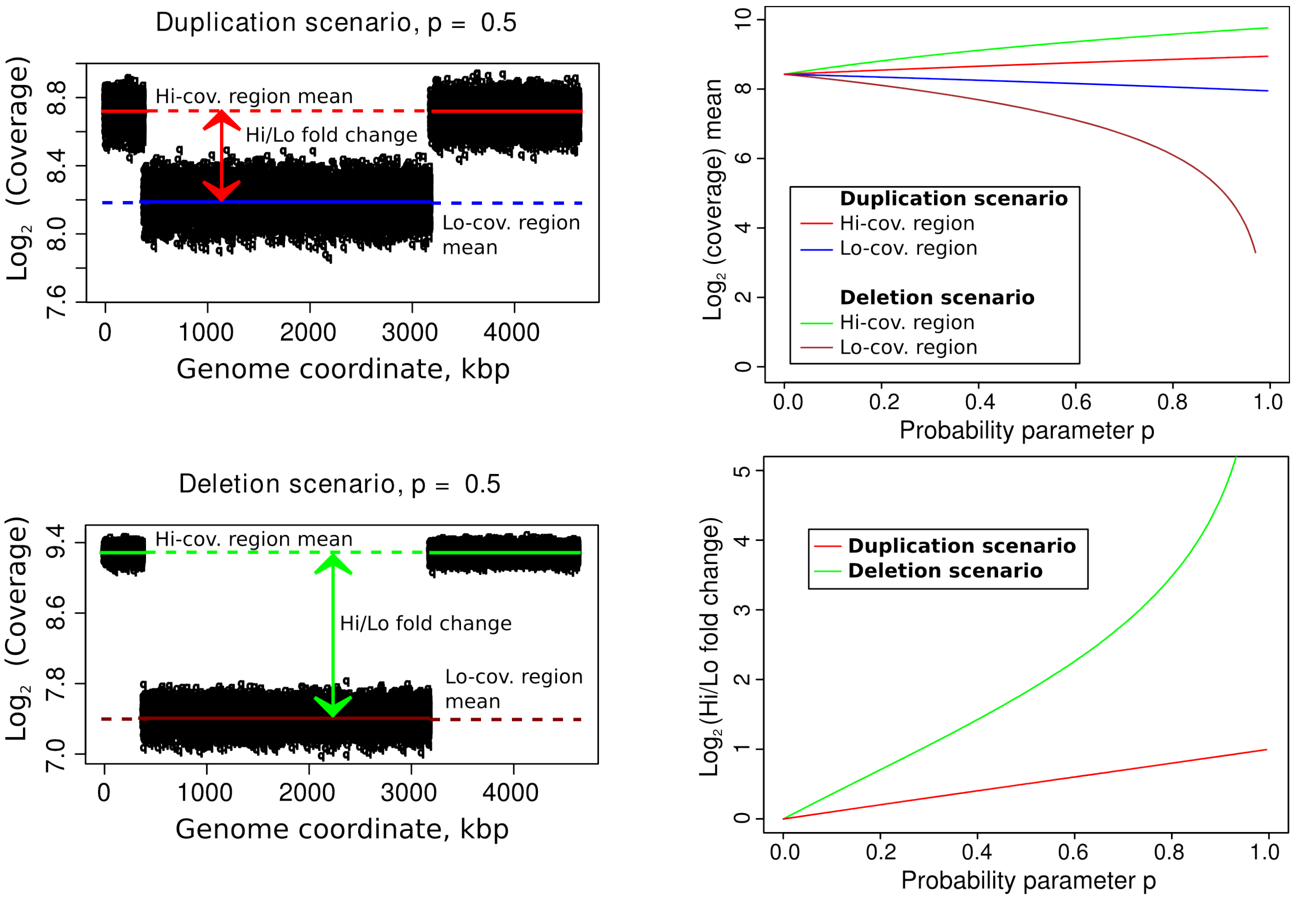

The two scatter plots on the left show the density of the randomized reads mapped to *E. coli* K12 chromosome for a chosen value of *p*. This is similar to the real experimental data shown in Figure 3 in the main text. The plots on the right show that the dependence of the mean coverage as a function of the mutated fraction *p* is different between the duplication and deletion scenarios. Note that parts of this figure are zoomed-in versions of smaller images shown in Supplementary Figure 8. In the deletion scenario the fold change between the two regions diverges more as a function of *p* than in the duplication scenario.

In the HS100 population the fold change in coverage between the High and the Low coverage regions has been measured at about ~1.2-fold, which according to our model would correspond to one of the two following scenarios: (a) a duplication in ~25% of the population; (b) a deletion in ~7% of the population. The number of HS100 single clones carrying the mutation (3 out of 16) is ~3-times more likely under the duplication scenario than with the deletion (by random sampling from 100s of clones).

A 1.8-fold change in coverage, as observed in a single colony sequencing, can be explained either by a deletion in ~25% of the population or an amplification in ~80% of the population. The fact that the proportion of cells with a deletion that can explain our observations is very low, also suggests that these cells will require an exceptional high rate of growth in order to result in an observable increase in population growth rates.

These together support the duplication model for the coverage data in HS100.

Supplementary Figure 10

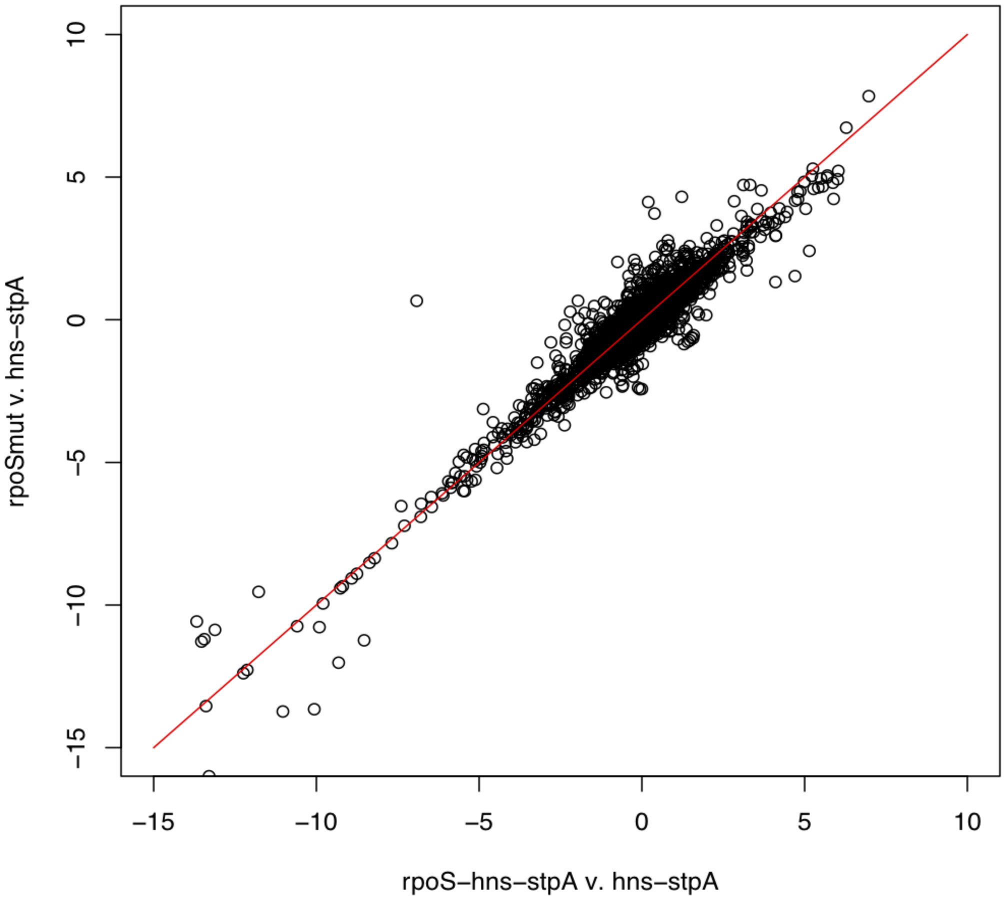

The above figure shows a scatter plot of the fold change in expression between the following comparison: x-axis, Δ*rpoS-hns-stpA* v. Δ*hns-stpA*; y-axis, *rpoS*^{mut} v. Δ*hns-stpA*. These indicate a high agreement between the two comparisons showing that much of the transcriptional effect seen in *rpoS*^{mut} can be explained by an *rpoS* knockout. All fold changes are in the log, base2 scale.

Supplementary Figure 11

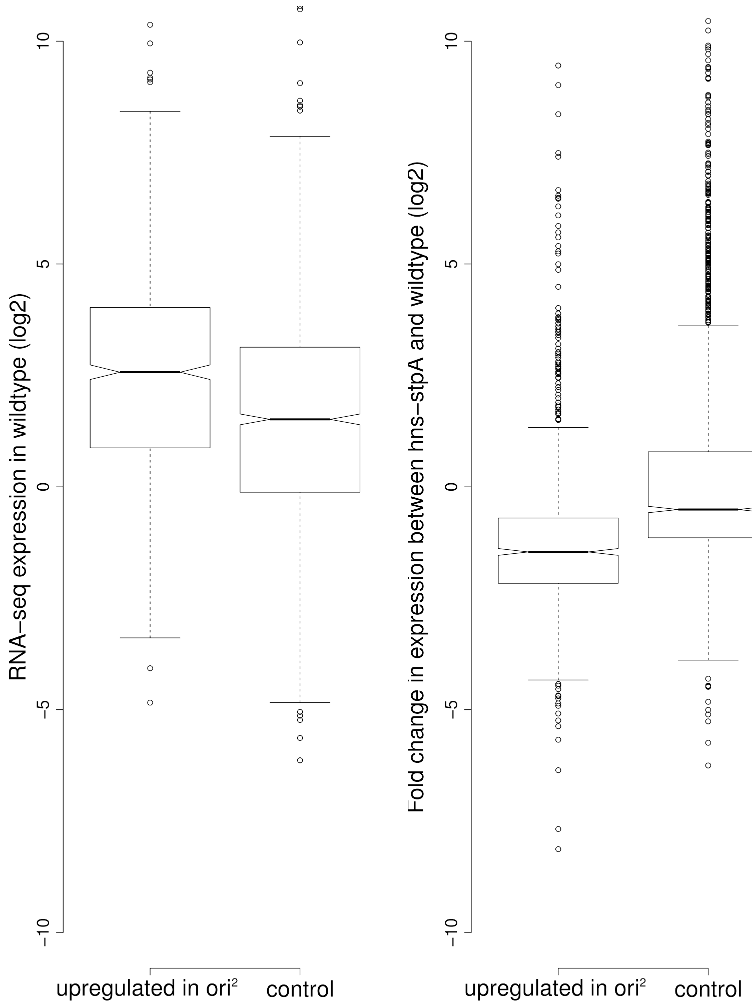

The above figure shows the gene expression properties of genes that are up-regulated in *ori²* relative to Δ*hns-stpA*. The left panel shows the wildtype mid-exponential phase expression levels of genes, which are up-regulated in *ori²*, and of a control set of genes, which do not change in expression in *ori²*. The right panel shows the fold change in expression between Δ*hns-stpA* and the wildtype for the same two sets of genes. Control genes were defined as those whose fold-change in expression between *ori²* and Δ*hns-stpA* were between -0.5 and +0.5 on the log2 scale. This figure shows that genes, which are up-regulated in *ori²* relative to Δ*hns-stpA* show higher-than-average expression levels in exponentially growing wildtype cells. Their expression is decreases in Δ*hns-stpA* when compared to the wildtype.

Supplementary Figure 12

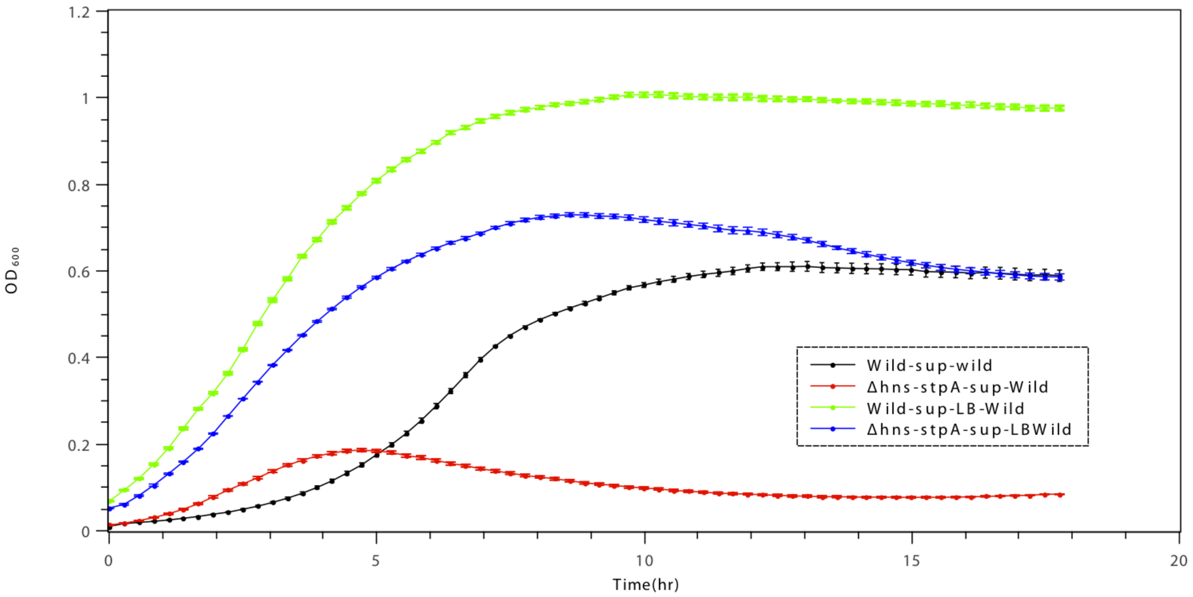

This figure shows the growth curve of wildtype *E. coli* grown in the spent media collected from the mid exponential phase culture of Δ*hns-stpA* (red) and wild type (black). The blue graph represents the growth of wild type *E. coli* in the Δ*hns-stpA* spent media supplemented with LB; and the green graph shows the growth of wild type *E. coli* grown in the wild type spent media supplemented with LB.

In rapidly-dividing cells, transient amplification of rRNA is common. Further, duplication of large segments of the chromosome can be selected in low nutrient environments. Polymorphisms in *rpoS* are common in both laboratory and environmental / pathogenic isolates of *E. coli*. Studies in chemostats have also shown that σ38 inactivation leads to shorter doubling times in low nutrient environments, and that such environments select for loss-of-function mutations in *rpoS*. We tested whether the *Δhns-stpA* culture might in fact be experiencing a 'low-nutrient' state. If this were the case, spent medium growing the double mutant to a certain cell density might support less growth than one in which the wildtype has grown to a similar cell density. We tested the ability of spent media, prepared from the wildtype and *Δhns-stpA* cultures, to support growth of the wildtype *E. coli* in 96-well plates (see figure). As expected, spent medium from a wildtype culture supported growth, albeit obviously to lower levels than fresh LB medium. On the other hand, medium from *Δhns-stpA* cultures supported less growth, in terms of both growth rate and final cell density. Further, the *Δhns-stpA* spent medium, when supplemented with LB constituents supported growth, which was comparable to that obtained from similar experiments with the wildtype supernatant. These suggest that *Δhns-stpA* cultures might be experiencing a low-nutrient state, which might be because of sub-optimal diversion of nutrients towards futile processes. This experience of nutrient limitation might be a common selective force for σ38 inactivation and duplication of the genomic domain around the origin of replication.

Supplementary Table 1a

Gene	Position	Substitution	Codon	Mutation	Description	References	Evolved samples Carrying the mutation
tauA	384965	I170I	ATT->ATA	T->A	TauA is the periplasmic substrate binding component of the taurine ABC transpoter	Ecocyc	**HS100_POP1** HS100_POP2 HS100G1 HS100G20
rhsD	523651	D389D	GAC->GAT	C->T	*rhsD* is one of the five homologous *rhs* loci that encode for a hydrophilic proteins with repetitive seqence. C-terminal domain of the protein is the toxic to the neighbouring cells.	Ecocyc	**HS100_POP1** HS100_POP2 HS100G1 HS100G20
etk	1041950	R495H	CGT->CAT	C->T	*E. coli* protein Tyrosine Kinase. Etk is required for the capsule formation in *E. coli*. Mutation in Etk and Etp affect the phospharytion and dephosphorylation cycles which are required for capsule formation.	C. Nadler *et al., PloS one*., (2012)	**HS100_POP1** HS100_POP2 HS100G1 HS100G20
fusA	3471131	P136S	CCG->TCG	G->A	Mutations in *fusA* give resistance to antibiotics	K.T.Lim *et al., Trans R Soc Trop Med Hyg*., (2014)	HS100_POP1 HS100_POP2 HS100G1 HS100G20
fusA	3471046	A164V	GCG->GTG	G->A			HS100_POP1 HS100_POP2 HS100G1 HS100G20
fusA	3469758	F593L	TTT->TTA	A->T			HS100G4 HS100G9
dusB	3409201	F300L	TTC->TTA	C->A	Gene codes for Dihydrouridine synthase B. Though *dusB* is co-transcribed with *fis*, *dusB* level in the cell is 0.5% as that of *fisA*.	Ecocyc	HS100G10
rpoS	2865113	I154N	ATT->AAT	A->T	Strains with non-functional RpoS or lower level of RpoS can scavenge nutrients better than the strains with functional RpoS and they have better competability.	T. King et al ., *J. Bacteriol*., (2004)	HS100_POP2 HS100G3
rpoS	2865447	E43*	GAG->TAG	C->A			HS100G5 HS100G11 HS100G13 HS100G15
rpoS	2865391	Y61*	TAC->TAG	G->C			HS100G12 HS100G16
ygbK	2861436	G360G	GGC->GGT	C->T	Unknown function		HS100G14
gadE	3656738	S117L	TCA->TTA	C->T	Glutamate dependent acid response gene regulate the expression of *gadA* and *gadBC* codes for glutamate decarboxylase . *gadE* is shown to be induced in the *hns* mutant	Z Ma *et al., J. Bacteriol*., (2004) F Hommais *et al., Mol. Microbiol*.., (2001)	HS100G17
lhr	1731048	R1313Q	CGG->CAG	G->A	Long Helicase related protein	Ecogene	HS100_POP1 HS100_POP2 HS100G1
lhr	1727609	R167S	CGC->AGC	C->A			HS100G17

dnaK	13270	A370T	GCT->ACT	G->A	*dnaK* mutants are defective in heat shock response.		HS100_POP2<br>HS100G1<br>HS100G20
erpA	176871	E88K	GAA->AAA	G->A	ErpA is an A-type Fe-S protein essential for the growth of E. coli in the presence of oxygen or alternative electron sources. Conserved cysteine in the residues such as 106, 42,108 are essential for the function of Erp protein	Loiseau *et al., Proc. Natl. Acad. Sci. U. S. A*..,(2007)	HS100_POP1<br>HS100_POP2<br>HS100G1<br>HS100G20
dnaE	205458	A111A	GCC->GCT	C->T	DnaE with GC->AT transition leads to the increased sensitivity to 2'-3' Dideoxy adenosine	K Hiratsuka *et al., J. Bacteriol*. (2001)	HS100_POP1<br>HS100_POP2<br>HS100G1<br>HS100G20
mhpF	372378	T78T	ACC->ACT	C->T	*mhpF* gene, encodes the acetylating NAD-dependent acetaldehyde dehydrogenase in *E. coli*. Δgpd1 and Δgpd2 are genes involved in the synthesis of NAD-dependent glycerol-3-phosphate dehydrogenase led to inability of the organism to grow under anaerobic condition, When mhpF was expressed in this mutant the organism can grow under anaerobic condition.	V. G. Medina *et al., Appl. Environ. Microbiol*., (2010)	HS100_POP1<br>HS100_POP2<br>HS100G1<br>HS100G20
ddlA	399424	S242C	AGC->TGC	T->A	*ddlA* codes for the D- alanine:D-alanine ligase. A365V mutation in *ddlA* gene of *Mycobacterium spegmatis* shows reduction in the enzymatic activity.	A.Belanger *et al., J. Bacteriol*., (2000)	HS100_POP1<br>HS100_POP2<br>HS100G1<br>HS100G20
sbcC	413481	Q499Q	CAG->CAA	C->T	Triple mutant lacking *recBC sbcB* are defective in growth and sensitive to Mitomycin C. Compensatory point mutation in *sbcC* restore the viability and resistance to Mitomycin C	R. Lloyd et al *J. Bacteriol*., (1985)	HS100_POP1<br>HS100_POP2<br>HS100G1<br>HS100G20
phoR	417352	H80H	CAC->CAT	C->T	Positive and negative regulatory gene for alkaline phosphatase and phosphodiesterase synthesis in *Bacillus subtilis*.	T. Seki *et al., J. Bacteriol*., (1988)	HS100_POP1<br>HS100_POP2<br>HS100G1<br>HS100G20
tesA	518536	P152S	CCC->TCC	G->A	Gene codes for Thioesterase I. Both deletion or overexpression of *tesA* had no effect on growth phenotype in *E. coli*	H Cho *et al., Biol Chem*., (1993)	HS100_POP1<br>HS100_POP2<br>HS100G1<br>HS100G20
asnB	697254	M383L	ATG->TTG	T->A	*asnB* encodes a structural protein involved in the biosynthesis of Asparagine and glutamate was induced by overexpression of acid response gene *gadX*	F Hommais *et al., Microbiology*., (2004)	HS100_POP1<br>HS100_POP2<br>HS100G1<br>HS100G20
sucC	762708	Y158N	TAT->AAT	T->A			HS100_POP1<br>HS100_POP2<br>HS100G1<br>HS100G20
dacC	880090	Y47*	TAC->TAA	C->A	*dacC* is a gene coding for penicillin binding protein 6 . *dacC* expression initiates in the stationary phase of	L. B. Pedersen *et al., J*	HS100_POP1<br>HS100_POP2

					growth cycle. Overexpression of DacC in *E.coli* is toxic.	*Bacteriol*., (1998)	HS100G1<br>HS100G20
trpB	1316141	R100Q	CGG->CAG	C->T	*trpA* and *trpB* are the genes codes for two components of the tryptophan synthase. Translation of *trpA* and *trpB* are coupled. In the absence of *trpB* translation *trpA* translation is not efficient.	S Aksoy *et al., J Bacteriol.,* (1984)	HS100_POP1<br>HS100_POP2<br>HS100G1<br>HS100G20
trpC	1316491	R440L	CGT->CTT	C->A	Gene product involved in tryptophan biosynthesis.	Ecogene	HS100_POP1<br>HS100_POP2<br>HS100G1<br>HS100G20
yciH	1340781	R34S	CGT->AGT	C->A	YciH binds 30S weakly and does not promote disassociation of subunits. YciH has some tRNA discriminatory activity in vitro; physiological role unknown. YciH also mimics some eIF1activities in vitro. Non-essential gene.	Ecogene	HS100_POP1<br>HS100_POP2<br>HS100G1<br>HS100G20
yciZ	1342543	N31Y	AAT->TAT	T->A	Unknown function		HS100_POP1<br>HS100_POP2<br>HS100G1<br>HS100G20
adhP	1551715	D50N	GAC->AAC	C->T	adhP gene product code for medium chain dehydrogenase/reductase activity.	Ecogene	HS100_POP1<br>HS100_POP2<br>HS100G1<br>HS100G20
ydeO	1581298	K138N	AAA->AAT	T->A	Under specific conditions *ydeO* activate the expression of the acid response genes *gadE.*	Z Ma *et al., J. Bacteriol*., (2004)	HS100_POP1<br>HS100_POP2<br>HS100G1<br>HS100G20
dmlA	1880567	E211V	GAG->GTG	A->T	*dmlA* codes for D-malate dehydrogenase an enzyme required for *E. coli* to grow under aerobic conditions on D- malate. Inactivation of the NAP such as FNR and ArcA cause decrease in the *dmlA* expression	H. Lukas *et al., J. Bacteriol*., (2010)	HS100_POP1<br>HS100_POP2<br>HS100G1<br>HS100G20
gabT	2790932	H59L	CAT->CTT	A->T	PuuE and GabT are redundant enzymes in putrescine catabolism	Ecogene	HS100_POP1<br>HS100_POP2<br>HS100G1<br>HS100G20
serA	3055394	R347C	CGT->TGT	G->A	*serA* codes for D-3-phosphoglycerate dehydrogenase, which catalyzes the first step in the biosynthesis of L-serine.	Ecocyc	HS100_POP1<br>HS100_POP2<br>HS100G1<br>HS100G20
waaA	3806982	A140A	GCG->GCT	G->T	*waaA* codes for 3-deoxy-D-manno-octulosonic acid (KDO) transferase (KdtA, WaaA) plays a key role in lipopolysaccharide biosynthesis.	Ecocyc	HS100_POP1<br>HS100_POP2<br>HS100G1<br>HS100G20
ibpA	3865348	G33D	GGC->GAC	C->T	*ibpA* is one of the stress response genes induced during the growth of the *E. coli* biofilm. *ibpAB* mutant shows reduced catalase activity and inhibiting the formation of biofilm at the air liquid interphase.	Kuczyńska-Wiśnik *et al., Microbiology.,* (2010)	HS100_POP1<br>HS100_POP2<br>HS100G1<br>HS100G20
pstB	3905885	A169T	GCC->ACC	C->T	*pstB* is one of a network of genes believed to play a role in promoting	Ecocyc	HS100_POP1<br>HS100_POP2

					the stress-induced mutagenesis (SIM) response of *E. coli* K-12		HS100G1<br>HS100G20
cadC	4359731	R76H	CGT->CAT	C->T	CadC a membrane integrated transcriptional activator indirectly senses the extracellular lysine by interacting with lysine permease Lys. CadC can't directly senses lysine as it has lower affinity to lysine.	L. Tetsch *et al.* ,*Mol. Microbiol.* (2008)	HS100_POP1<br>HS100_POP2<br>HS100G1<br>HS100G20
cspC	1905408	G18S	GGC->AGC	A->G	CspC mutant with G18R substitution shows increased fitness .<br>CspC is induced by the glutamate dependent acid response gene GadE and GadX.	D. Rath *et al.*, *J. Bacteriol. (2006)*<br><br>F Hommais *et al .*, *Microbiology.*, (2004)	**HS250_POP1**<br>HS250G1<br>HS250G5<br>HS250G7<br>HS250G8
yeiB	2239953	L346P	CTG->CCG	C->T	Unknown function		**HS250_POP1**<br>HS250G1<br>HS250G5<br>HS250G7<br>HS250G8
recC	2958797	E552K	GAA->AAA	G->A	*recC* mutants shows decreased P1-transduction ability, lesses recombination efficiency and increased sensitivity to UV.	S. T Lovett *et al .*, *Genetics.*, (1988)	**HS250_POP1**<br>HS250G1<br>HS250G5<br>HS250G7<br>HS250G8
kduI	2981483	Q222*	CAG->TAG	T->C	KduI and KduD facilitates the breakdown of hexouronate. When grown under the hexouronates such as Galactouronate and Glucouronates *kduID* deficient cells had 30-80% lower maximal cell density and 1.5-2 fold longer doubling time than the wild type cells.	M. Rothe *et al., PLOS one.*, (2013)	**HS250_POP1**<br>HS250G1<br>HS250G5<br>HS250G7<br>HS250G8
ygfS	3026707	T110A	ACC->GCC	T->C	Putative electron transport protein	Uniprot	**HS250_POP1**<br>HS250G1<br>HS250G5<br>HS250G7<br>HS250G8
tatC	4021452	F232L	TTC->CTC	C->T	TatC is a subunit of the TatABCE (twin-arginine translocation) complex for the export of folded proteins across the cytoplasmic membrane. *tatC* is one of a network of genes believed to play a role in promoting the stress-induced mutagenesis (SIM) response of *E. coli* K-12	Ecocyc	**HS250_POP1**<br>HS250G1<br>HS250G5<br>HS250G7<br>HS250G8
mscM	4385295	R700K	AGG->AAG	C->G	MscM is a mechanosensitive ion channel of miniconductance in *E. coli*	Ecocyc	**HS250_POP1**<br>HS250G1<br>HS250G5<br>HS250G7<br>HS250G8
mutL	4397069	Y545*	TAC->TAG	G->A	High copy number of MutL encoded by *mutL* gene shows decrease in the mutation rate. High copy number of	Galán, J.-C *et al., FEMS*	**HS250_POP1**<br>HS250G1<br>HS250G5

					MutL can also partially revert the hypermutator phenotype MutS defective strain of *E. coli*	*Microbiol. Lett.*(2007)	HS250G7 HS250G8
gldA	4136555	G168G	GGT->GGC	T->C	GldA was involved in glycerol fermentation both as a glycerol dehydrogenase, producing dihydroxyacetone, and as a 1,2-propanediol dehydrogenase, regenerating NAD^+ by producing 1,2-propanediol from acetone	Ecocyc	HS250_POP1 HS250G7
uxuR	4552975	R126H	CGT->CAT	G->A	UxuR is important for the growth of *E. coli* on fructuronate.	C Utz *et al* ., *J. Bacteriol*., (2004)	HS250_POP1 HS250G7
frsA	257728	G401D	GGT->GAT	G->A	FrsA maintains a flux between respiration and fermentation in *E. coli*. Disruption of *frsA* increases cellular respiration on several sugars including glucose and while, increased *frsA* expression resulted in an increased fermentation on sugars.	Koo, B.-M *et al., J. Biol. Chem*. (2004)	HS250_POP1 HS250G7
aslA	3982512	L507F	CTC->TTC	G->A	The gene *aslA* codes for a latent arylsulfatase protein in *E. coli*. It is expressed only when the adjacent tyramine oxidase gene is induced and is not directly controlled by the sulfur supply		**HS250_POP2** HS250_POP1 HS250G8
rplF	3443822	D114G	GAC->GGC	A->G	50S ribosomal subunit protein L6	Ecogene	HS250G1
valS	4479297	V855A	GTG->GCG	G->A	Valine t-RNA synthetase	Ecogene	HS250G1
moeA	865104	P162S	CCA->TCA	T->C	molybdopterin molybdenumtransferase; molybdopterin biosynthesis protein	Ecogene	HS250G1
eutC	2554837	V161V	GTA->GTG	A->G	ethanolamine ammonia-lyase, small subunit (light chain)	Ecogene	HS250G1
yacC	136755	I55V	ATT->GTT	A->G	conserved protein, PulS_OutS family	Ecogene	HS250G5
yahN	345053	V170A	GTG->GCG	G->A	amino acid exporter for proline, lysine, glutamate, homoserine	Ecogene	HS250G5
ynfF	1659839	W420*	TGG->TGA	A->G	S- and N-oxide reductase, A subunit, periplasmic	Ecogene	HS250G5
tldD	3389253	G266G	GGT->GGC	T->C	Putative peptidase	Ecogene	HS250G5
yhiM	3632887	L8L	CTT->CTC	C->T	Acid resistance protein, inner membrane	Ecogene	HS250G5
mdtH	1123756	R265H	CGC->CAC	A->G	Multidrug resistance efflux transporter conferring overexpression resistance to norfloxacin and enoxacin	Ecogene	HS250G5
pdxA	53093	C108C	TGT->TGC	C->T	4-hydroxy-L-threonine phosphate dehydrogenase, NAD-dependent	Ecogene	HS250G5
yjeH	4367513	R308H	CGC->CAC	T->C	Putative transporter	Ecogene	HS250G5
yfcH	2419825	R32R	CGT->CGC	T->C	Conserved protein with NAD(P)-binding Rossmann-fold domain	Ecogene	HS250G5
bamA	198547	N207S	AAC->AGC	A->G	Outer membrane protein assembly factor, forms pores; required for OM biogenesis; in BamABCDE OM protein complex	Ecogene	HS250G7
ydiN	1771040	G169R	GGG->AGG	G->A	Inner membrane protein, predicted MFS superfamily transporter	Ecogene	HS250G7

rcsD	2313086	L526P	CTG->CCG	T->C	Phosphotransfer intermediate protein in two-component regulatory system with RcsBC	Ecogene	HS250G7
barA	2914521	G481G	GGC->GGT	C->T	Hybrid sensory histidine kinase, in two-component regulatory system with UvrY	Ecogene	HS250G7
fadB	4028262	G245R	GGG->AGG	C->T	Fused 3-hydroxybutyryl-CoA epimerase/delta(3)-cis-delta(2)-trans-enoyl-CoAisomerase/enoyl-CoAhydratase/3-hydroxyacyl-CoA dehydrogenase	Ecogene	HS250G7
mscM	4385281	V705I	GTT->ATT	C->T	Mechanosensitive channel protein, miniconductance	Ecogene	HS250G7
hypF	2834392	N352N	AAC->AAT	G->A	Carbamoyl phosphate phosphatase and maturation protein for [NiFe] hydrogenases	Ecogene	HS250G8
yhgN	3573585	G164G	GGC->GGT	C->T	Putative antibiotic transporter	Ecogene	HS250G8
metA	4213131	W277R	TGG→CGG	T->C	Homoserine O-transsuccinylase	Ecogene	HS250G8
recB	2951465	R854H	CGC→CAC	C->T	Exonuclease V (RecBCD complex), beta subunit	Ecogene	HS250G8
polB	64351	R477H	CGC→CAC	C->T	DNA polymerase II	Ecogene	HS250G8

Table shows the SNPs found in the evolved strains. SNPs seen in the evolved population samples with Variance Frequency (VF) of >= 20 % are highlighted in the bold letters. Other population samples shows the mutation with lesser frequency.

References

1. Belanger, A.E., Porter, J.C. and Hatfull, G.F. (2000) Genetic Analysis of Peptidoglycan Biosynthesis in Mycobacteria : Characterization of a ddlA Mutant of Mycobacterium smegmatis Genetic Analysis of Peptidoglycan Biosynthesis in Mycobacteria : Characterization of a ddlA Mutant of Mycobacterium smegmatis. 10.1128/JB.182.23.6854-6856.2000.Updated.

2. Chan, F. and Torriani, A. (1996) PstB protein of the phosphate-specific transport system of Escherichia coli is an F Y Chan and A Torriani These include : PstB Protein of the Phosphate-Specific Transport System of Escherichia coli Is an ATPase Downloaded from http://jb.asm.org/ on June 2.

3. Cho, H. and Cronan, J.E. (1993) Escherichia coli thioesterase I, molecular cloning and sequencing of the structural gene and identification as a periplasmic enzyme. *J. Biol. Chem.*, **268**, 9238–45.

4. Galán, J.-C., Turrientes, M.-C., Baquero, M.-R., Rodríguez-Alcayna, M., Martínez-Amado, J., Martínez, J.-L. and Baquero, F. (2007) Mutation rate is reduced by increased dosage of mutL gene in Escherichia coli K-12. *FEMS Microbiol. Lett.*, **275**, 263–9.

5. Guadalupe Medina, V., Almering, M.J.H., van Maris, A.J. a and Pronk, J.T. (2010) Elimination of glycerol production in anaerobic cultures of a Saccharomyces cerevisiae strain engineered to use acetic acid as an electron acceptor. *Appl. Environ. Microbiol.*, **76**, 190–5.

6. Hiratsuka, K. and Reha-krantz, L.J. (2000) Identification of Escherichia coli dnaE (polC) Mutants with Altered Sensitivity to 2 ′ , 3 ′ Identification of Escherichia coli dnaE (polC) Mutants with Altered Sensitivity to 2 J , 3 J -Dideoxyadenosine. **182**.

7. Hommais, F. (2004) GadE (YhiE): a novel activator involved in the response to acid environment in Escherichia coli. *Microbiology*, **150**, 61–72.

8. Hommais, F., Krin, E., Laurent-Winter, C., Soutourina, O., Malpertuy, a, Le Caer, J.P., Danchin, a and Bertin, P. (2001) Large-scale monitoring of pleiotropic regulation of gene expression by the prokaryotic nucleoid-associated protein, H-NS. *Mol. Microbiol.*, **40**, 20–36.

9. Javaux, C., Joris, B. and De Witte, P. (2007) Functional characteristics of TauA binding protein from TauABC Escherichia coli system. *Protein J.*, **26**, 231–8.

10. King, T., Ishihama, A., Kori, A. and Ferenci, T. (2004) A Regulatory Trade-Off as a Source of Strain Variation in the Species Escherichia coli A Regulatory Trade-Off as a Source of Strain Variation in the Species Escherichia coli †. **186**.

11. Koo, B.-M., Yoon, M.-J., Lee, C.-R., Nam, T.-W., Choe, Y.-J., Jaffe, H., Peterkofsky, A. and Seok, Y.-J. (2004) A novel fermentation/respiration switch protein regulated by enzyme IIAGlc in Escherichia coli. *J. Biol. Chem.*, **279**, 31613–21.

12. Kuczyńska-Wiśnik, D., Matuszewska, E. and Laskowska, E. (2010) Escherichia coli heat-shock proteins IbpA and IbpB affect biofilm formation by influencing the level of extracellular indole. *Microbiology*, **156**, 148–57.

13. Lloyd, R.G. and Buckman, C. (1985) Identification and genetic analysis of sbcC mutations in commonly used recBC sbcB strains of Escherichia coli K-12. *J. Bacteriol.*, **164**, 836–44.

14. Loiseau, L., Gerez, C., Bekker, M., Ollagnier-de Choudens, S., Py, B., Sanakis, Y., Teixeira de Mattos, J., Fontecave, M. and Barras, F. (2007) ErpA, an iron sulfur (Fe S) protein of the A-type essential for respiratory metabolism in Escherichia coli. *Proc. Natl. Acad. Sci. U. S. A.*, **104**, 13626–31.

15. Lovett, S.T., Luisi-deluca, C. and Kolodner, R.D. (1988) Genetic dependence of recombination in the recD mutants of *Escherichia coli*. *Genetics.,* **120,** 37-45.

16. Lukas, H., Reimann, J., Kim, O. Bin, Grimpo, J. and Unden, G. (2010) Regulation of aerobic and anaerobic D-malate metabolism of Escherichia coli by the LysR-type regulator DmlR (YeaT). *J. Bacteriol.*, **192**, 2503–11.

17. Ma, Z., Masuda, N. and Foster, J.W. (2004) Characterization of EvgAS-YdeO-GadE Branched Regulatory Circuit Governing Glutamate-Dependent Acid Resistance in Escherichia coli. **186**, 7378–7389.

18. Nadler, C., Koby, S., Peleg, A., Johnson, A.C., Suddala, K.C., Sathiyamoorthy, K., Smith, B.E., Saper, M. a and Rosenshine, I. (2012) Cycling of Etk and Etp phosphorylation states is involved in formation of group 4 capsule by Escherichia coli. *PLoS One*, **7**, e37984.

19. Park, S. and Chao, G. (1997) Aerobic regulation of the sucABCD genes of Escherichia coli , which encode alpha-ketoglutarate dehydrogenase and succinyl coenzyme A synthetase : roles of ArcA , Fnr , and the upstream sdhCDAB promoter . Aerobic Regulation of the sucABCD Genes of Escheric.

20. Pedersen, L.B., Murray, T., Popham, D.L. and Setlow, P. (1998) Characterization of dacC, which encodes a new low-molecular-weight penicillin-binding protein in Bacillus subtilis. *J. Bacteriol.*, **180**, 4967–73.

21. Pratt, C. and Gallant, J. (1972) A DOMINANT CONSTITUTIVE phoR MUTATION IN ESCHERICHIA COLP exercised primarily by two loci , designated phoR and phoS . Both are located and ECHOLS and ECHOLS (1962) to propose a model for the types of mutations led GAREN regulation of alkaline phosphata.

22. Rothe, M., Alpert, C., Loh, G. and Blaut, M. (2013) Novel Insights into E . coli ' s Hexuronate Metabolism : KduI Facilitates the Conversion of Galacturonate and Glucuronate under Osmotic Stress Conditions. **8**.

23. Rath, D. and Jawali, N. (2006) Loss of expression of cspC, a cold shock family gene, confers a gain of fitness in Escherichia coli K-12 strains. *J. Bacteriol.*, **188**, 6780–5.

24. Scofield, M. a, Lewis, W.S. and Schuster, S.M. (1990) Nucleotide sequence of Escherichia coli asnB and deduced amino acid sequence of asparagine synthetase B. *J. Biol. Chem.*, **265**, 12895–902.

25. Seki, T., Yoshikawa, H., Takahashi, H. and Saito, H. (1988) Nucleotide sequence of the Bacillus subtilis Nucleotide Sequence of the Bacillus subtilis phoR Gene. **170**.

26. Tetsch, L., Koller, C., Haneburger, I. and Jung, K. (2008) The membrane-integrated transcriptional activator CadC of Escherichia coli senses lysine indirectly via the interaction with the lysine permease LysP. *Mol. Microbiol.*, **67**, 570–83.

27. Utz, C.B., Nguyen, A.B., Smalley, D.J., Anderson, B., Conway, T. and Anderson, A.B. (2004) GntP Is the Escherichia coli Fructuronic Acid Transporter and Belongs to the UxuR Regulon GntP Is the Escherichia coli Fructuronic Acid Transporter and Belongs to the UxuR Regulon. 10.1128/JB.186.22.7690.

Supplementary Table 1b

Gene	Position	Mutation	Annotation	Description	References	Evolved samples Carrying the indel
trpB	1316412	+A	coding (28/1194 nt)	Tryptophan synthase, beta subunit.	Ecogene	HS100G1 HS100G20
paaY	1462590	+A	coding (96/591 nt)	Putative hexapeptide repeat acetyltransferase	Ecogene	HS100G1 HS100G20
bcsC	3685025	+A	coding (2172/3474 nt)	Cellulose synthase subunit	Ecogene	HS100G1 HS100G20
rpoS	2864709	2 bp→AA	coding (864-865/993nt)	RNA polymerase, sigma S (sigma 38) factor. Strains with non-functional RpoS or lower level of RpoS can scavenge nutrients better than the strains with functional RpoS and they have better competability.	T. King *et al.*, *J. Bacteriol.*, (2004)	HS100G14
rcsB	2314694	Δ1 bp	coding (496/651 nt)	DNA-binding positive regulatory gene for capsule synthesis. GadE and phosphorylated RcsB form a protein complex to regulate glutamate-, arginine- and lysine-dependent acid resistance	Ecogene	HS250G1 HS250G8 HS250G5 HS250G7
appY	582979	Δ1 bp	coding (76/750 nt)	DNA-binding global transcriptional activator; DLP12 prophage. H-NS negatively regulates the expression of the activator AppY.	A.Tove *et al., J Bacteriol.,* (1996)	HS250G8 HS250G5 HS250G7
leuQ	4604345	+G	noncoding (80/87 nt)	tRNA-Leu	Ecogene	HS250G5
glcF	3123436	Δ2 bp	coding (45-46/1224 nt)	Glycolate oxidase 4Fe-4S iron-sulfur cluster subunit. he insertional mutation by a chloramphenicol acetyltransferase cassette in either glcD,glcE,or glcF abolished glycolate oxidase activity	M. Pellicer *et al., J Bacteriol.*, (1996)	HS250G5
hycD	2844639	Δ1 bp	coding (796/924 nt)	Hydrogenase 3, membrane subunit	Ecogene	HS250G8
zapB	4116637	+A	coding (100/246 nt)	FtsZ stabilizer; septal ring assembly factor, stimulates	E. Galli *et al., J Bacteriol.*, (2012)	HS250G7

				cell division. ZapB interacts strongly with ZapA and involved in FtsZ ring formation		
smf	3431010	+C	coding (573/1125 nt)	Hypothetical protein	Ecogene	HS250G7
tsx	431227	+T	coding (11/885 nt)	Nucleoside channel, receptor of phage T6 and colicin K. Tsx – a porin protein is positively regulated by H-NS. Tsx expression is decreased on inactivation of H-NS	P. Landini *et al., J Bacteriol.,* (2002)	HS250G5
yfjQ	2767139	Δ1 bp	coding (453/822 nt)	CP4-57 prophage; putative protein	Ecogene	HS250G8

References

1. Landini, P. and Zehnder, A.J.B. (2002) The Global Regulatory hns Gene Negatively Affects Adhesion to Solid Surfaces by Anaerobically Grown Escherichia coli by Modulating Expression of Flagellar Genes and Lipopolysaccharide Production. **184**, 1522–1529.

2. Atlung, T., Sund, S., Olesen, K., Brøndsted, L., Atlung, T., Sund, S. and Olesen, K. (1996) The histone-like protein H-NS acts as a transcriptional repressor for expression of the anaerobic and growth phase activator AppY of Escherichia coli . The Histone-Like Protein H-NS Acts as a Transcriptional Repressor for Expression of the Anaerobic and Growth Phase Activator AppY of Escherichia coli.

3. Galli, E. and Gerdes, K. (2012) FtsZ-ZapA-ZapB interactome of Escherichia coli. *J. Bacteriol*., **194**, 292–302.

4. King, T., Ishihama, A., Kori, A. and Ferenci, T. (2004) A Regulatory Trade-Off as a Source of Strain Variation in the Species Escherichia coli A Regulatory Trade-Off as a Source of Strain Variation in the Species Escherichia coli †. **186**.

5. Pellicer, M.T., Badía, J., Aguilar, J. and Baldomà, L. (1996) glc locus of Escherichia coli : characterization of genes encoding the subunits of glycolate oxidase and the glc regulator protein . These include : glc Locus of Escherichia coli : Characterization of Genes Encoding the Subunits of Glycolate Oxidase and the glc Regulator Protein. *J. Bacteriol*., **178**.

6. http://www.ecogene.org/

Supplementary table 2

Comparison	***rpoS*^{mut} *and Δhns-stpA***		***Δhns-stpA-rpoS and Δhns-stpA***		***Wild- Δhns-stpA***	
**Method**	RT-PCR	RNAseq	RT-PCR	RNA seq	RT-PCR	RNAseq
**Genes**						
rssA	0.988±0.180	0.89528567	0.709±0.047	0.82462494	1.524±0.293	1.335611111
rssB	1.048±0.162	0.398623868	0.813±0.111	0.417688695	3.754±0.712	1.253967537
katE	0.180±0.044	0.065389373	0.097±0.013	0.073220935	0.472±0.081	0.232854392
OtsA	0.126±0.058	0.156978621	0.047±0.001	0.195426769	0.703±0.035	0.637015331
rpoS	1.298±0.282	1.583755907	0.018±0.006	0.008269983	2.4550±0.565	4.967286638
aldB	0.466±0.170	0.158691856	0.247±0.018	0.177969263	5.91±1.36	2.676411596

Supplementary Table 3

Name	Primer sequence
**Primers used for the generation of knockouts**	
hns-KO-fwd	CCTCAACAAACCACCCCAATATAAGTTTGAGATTACTACAGTGTAGGCTGGAGCTGCTTCG
hns-KO-rev	GCCGCTGGCGGGATTTTAAGCAAGTGCAATCTACAAAAGACATATGAATATCCTCCTTA
rpoS-KO-fwd	TTGAATGTTCCGTCAAGGGATCACGGGTAGGAGCCACCTTGTGTAGGCTGGAGCTGCTTC
rpoS-KO-rev	CCAGCCTCGCTTGAGACTGGCCTTTCTGACAGATGCTTACCCGGGGATCCGTCGACC
stpA-KO-fwd	CTTTTTTTGTTTTGGCGTTAAAAGGTTTTCTTTATTGTGTAGGCTGGAGCTGCTTC
stpA-KO rev	CGGACGCGCCCTAGCAGCGACATCCGGCCTCAGTAACCGGGGATCCGTCGACC
**External primers used for the detection of knockouts**	
hns-ext-fwd	CCTCAACAAACCACCCCAATATAAGTTTGAGATTACTACA
hns-ext-rev	GCCGCTGGCGGGATTTTAAGCAAGTGCAATCTACAAAAGA
rpoS-ext-fwd	TTGAATGTTCCGTCAAGGGATCACGGGTAGGAGCCACCTT
rpoS-exr-rev	CCAGCCTCGCTTGAGACTGGCCTTTCTGACAGATGCTTAC
stpA-ext-fwd	AATACTTTTTTTGTTTTGGCGTTAAAAGGTTTTCTTTATT
stpA-ext-rev	ACGCCGGACGCGCCCTAGCAGCGACATCCGGCCTCAGTAA
**Internal primers used for the confirmation of knockouts**	
hns-int-fwd	GGCTGCTGCTGAAGTTGAAG
hns-int-rev	GTTTCGCCGTTTTCGTCAACG
stpA-int-fwd	CCTCCGTGCGATGGCTCGCG
stpA-int- rev	GAATTTATATTTCGCCGGACGCG
Kan-int-fwd	CGGTGCCCTGAATGAACTGC
Kan-int-rev	CGGCCACAGTCGATGAATCC
rpoS_snp_Fwd	CAGCAAAGGACAGGCAATTATC
rpoS_snp_rev	GTAAACGTTCAGCTCCTTTACGA
lhr_R167S_Fwd	GGCAGATAATCCAGACCCTTC
lhr_R167S_rev	ATCGTGCCGTCAGTTTTTCC
lhr_R1313Q_Fwd	CGAAGAATCTAACCAGACCATCA
lhr_R1313Q_Rev	CAGAAGATTTCCCCACACATTAG
FusA_(F593L)_Fwd	TCCGTGTATGGACTGACGAA
FusA_(F593L)_Rev	ACTCGGCGCTTCATCATACT
appY_del1bp_Fwd	TCAGGTGCGTTGTAGTGAGTTT
appY_del1bp_Rev	CAGTAAATGACGTTCCTTCATCC
GadE_S117L_Fwd	GAACAACGATTCGGACAAG
GadE_S117L_Rev	GTGGGATACAGGCACAGTGAT
CspC_G18S_Rev	CTGAACTGGCAAGGTTAAGCAC
CspC_G18S_Fwd	ATGTCGCTGGGGACAGATATAG
RecC_E552K_Fwd	GCTGATATCGACAGCTACAGTCC
RecC_E552K_Rev	ACGCTTCCAGGAACAGATAGC
rpoS-2bp_ins_Fwd	TTATCGAAGAGGGCAACCTG
rpoS-2bp_ins_Rev	ACAGCGCTTCGATATTCAGC
MutL_Y545*_Fwd	GCGAACAGTCAGAGTTTTGGTC
MutL_Y545*_Rev	CGAGTAACTCTTCAGCGTTCG

**RpoS targets used for RT-PCR validation**	
rssB-RT-fwd	TGGGAGGTTTCACTCCAGAC
rssB-RT-Rev	AACATCTTCAACGCCCAGAC
katE-RT-fwd	AATTCCACAAGGGCAAAGTG
katE-RT-rev	CTTCCCTTCGGCATTAATCA
aldB-fwd	AGATTAAGCCCGGCGAGTAT
aldB-rev	GCCAGATCGATGTCTCGTTT
rssA-RT-fwd	CCGGAAACAGAGATCGAAAA
rssA–RT-rev	ACCAGCCAGTAGCCGTTATG
otsA-RT-fwd	GACTGTGGTTTGGCTGGAGT
otsA-RT-rev	CACCAGATCGAGCCGATAAT
